# Fish conservation in the land of steppe and sky: Evolutionarily significant units of threatened salmonid species in Mongolia mirror major river basins

**DOI:** 10.1002/ece3.4974

**Published:** 2019-02-27

**Authors:** Andrew Kaus, Stefan Michalski, Bernd Hänfling, Daniel Karthe, Dietrich Borchardt, Walter Durka

**Affiliations:** ^1^ Department of Aquatic Ecosystem Analysis and Management Helmholtz Centre for Environmental Research – UFZ Magdeburg Germany; ^2^ Department of Agriculture and Fisheries Bribie Island Research Centre Woorim Australia; ^3^ Department of Community Ecology Helmholtz Centre for Environmental Research – UFZ Halle Germany; ^4^ School of Environmental Sciences University of Hull Hull UK; ^5^ Environmental Engineering Section German Mongolian Institute for Resources and Technology Nalaikh Mongolia; ^6^ German Centre for Integrative Biodiversity Research (iDiv) Halle‐Jena‐Leipzig Leipzig Germany

**Keywords:** *Brachymystax lenok*, evolutionarily significant units, freshwater fish conservation, *Hucho taimen*, threatened salmonids, *Thymallus baicalensis*

## Abstract

Mongolia's salmonids are suffering extensive population declines; thus, more comprehensive fisheries management and conservation strategies are required. To assist with their development, a better understanding of the genetic structure and diversity of these threatened species would allow a more targeted approach for preserving genetic variation and ultimately improve long‐term species recoveries. It is hypothesized that the unfragmented river basins that have persisted across Mongolia provide unobstructed connectivity for resident salmonid species. Thus, genetic structure is expected to be primarily segregated between major river basins. We tested this hypothesis by investigating the population structure for three salmonid genera (*Hucho, Brachymystax* and *Thymallus*) using different genetic markers to identify evolutionarily significant units (ESUs) and priority rivers to focus conservation efforts. Fish were assigned to separate ESUs when the combined evidence of mitochondrial and nuclear data indicated genetic isolation. *Hucho taimen* exhibited a dichotomous population structure forming two ESUs, with five priority rivers. Within the *Brachymystax* genus, there were three *B. lenok*ESUs and one *B*. *tumensis*ESU, along with six priority rivers. While *B*. *tumensis*was confirmed to display divergent mtDNA haplotypes, haplotype sharing between these two congeneric species was also identified. For *T. baicalensis,*only a single ESU was assigned, with five priority rivers identified plus Lake Hovsgol. Additionally, we confirmed that *T. nigrescens* from Lake Hovsgol is a synonym of *T. baicalensis*. Across all species, the most prominent pattern was strong differentiation among major river basins with low differentiation and weak patterns of isolation by distance within river basins, which corroborated our hypothesis of high within‐basin connectivity across Mongolia. This new genetic information provides authorities the opportunity to distribute resources for management between ESUs while assigning additional protection for the more genetically valuable salmonid rivers so that the greatest adaptive potential within each species can be preserved.

## 1 INTRODUCTION

Mongolia's vast river basins include some of the least impacted freshwater ecosystems on the planet (Hofmann et al., 2015). However, across many regions, a multitude of anthropogenic pressures is currently threatening this pristine status, with increasing contamination and degradation of aquatic environments and their resident species (Stubblefield et al., 2005; Karthe et al., 2017). The damage to Mongolia's rivers, streams, and lakes has been a direct result of recent, rapid development, where high rates of urbanization and industrialization have led to growing discharge of poorly treated wastewater, increased industrial pollution and mining contamination, along with rising rates of overgrazing and deforestation in many river basins (Hartwig et al., 2016; Hofmann, Venohr, Behrendt, & Opitz, 2010; Kaus, Schäffer et al., 2016). However, the main driver of many native fish population declines, including a number of salmonid species that have suffered regional losses suspected of being up to 50% in recent decades, has been intensifying fishing activities, which has continued to gain rapid popularity across the country (Chandra, Gilroy, Purevdorj, & Erdenebat, 2005; Hogan & Jensen, 2013; Kaus, 2018; Ocock et al., 2006). In order to help mitigate these declines, improvements to the current fisheries management strategies are required, with an essential first step being the identification of ecologically meaningful management units across the wider distribution of each target species so authorities and policy makers can better understand the functional scale of the threatened populations that they are trying to manage and conserve (Funk, McKay, Hohenlohe, & Allendorf, 2012).

Evolutionarily significant units (ESUs) are a common management tool used in conservation biology that involves the identification of intraspecific groups which represent a more biologically meaningful assemblage within a species' geographic distribution. While a number of important factors are typically considered in order to define species' ESUs, including ecologically relevant phenotypic attributes or certain life history traits; one of the more unequivocal techniques that can determine a measurable divergence between isolated groups of conspecifics has been modern genetic methods (Avise, 2000; Bernard et al., 2009; Fraser & Bernatchez, 2001). ESUs can consist of multiple allopatric populations and can cover extensive geographic regions depending on the species and its ecology (Moritz, 1994; Palsbøll, Bérube, & Allendorf, 2006). Units are usually defined based on neutral and sometimes adaptive genetic variation, which represent the effects of both historical spatial processes and environmental selection (Casacci, Barbero, & Balletto, 2014; Crandall, Bininda‐Emonds, Mace, & Wayne, 2000; Funk et al., 2012; Moritz, 1994). For the initial demarcation of an ESU, researchers have focused on genetic markers including maternally transmitted, slowly evolving mtDNA, but also biparentally transmitted, quickly evolving microsatellites, as both yield relevant information on complementary spatiotemporal scales (O'Connell & Wright, 1997; Vogler & DeSalle, 1994). The identification of ESUs and genetically distinct populations of threatened and exploited fish stocks is increasingly used in fishery management to ensure that conservation actions and resources can be better matched with biological relevance (Xia, Chen, & Sheng, 2006; Geist, Kolahsa, Gum, & Kuehn, 2009; Escobar, Andrade‐López, Farias, & Hrbek, 2015; Zhivotovsky et al., 2015). While ESUs represent the upper hierarchical levels of intraspecific biodiversity, demographically independent groups that harbor an above average genetic variation or are more genetically distinct compared to the rest of the ESU are also important to identify. With recent studies reporting a strong association between alleles at one or a very few genes and a key life history trait in Pacific salmonid species (Hess, Zendt, Matala, & Narum, 2016; Prince et al., 2017), knowledge of these more genetically valuable or priority populations and their geographic extent, i.e. their river system, may be increasingly important to identify for the conservation of other threatened salmonids as well. Such information can assist in designing adequate protection and recovery programs for threatened species, as conservation efforts can thus focus on preserving the ability of natural ecological and evolutionary processes which produce genetic variation capable of sustaining a species long term under future shifting environmental conditions (Petit & Mousadik, 1998; Waples & Lindley, 2018).

Mongolia's salmonids species from the genera *Hucho, Brachymystax,*and *Thymallus* (Family *Salmonidae*) live in sympatry throughout the country's two major river basins. While there are still some remote rivers systems holding robust numbers of these species, widespread declines have continued throughout much of their range both in Mongolia and neighboring Russia and China (Hogan & Jensen, 2013; Ocock et al., 2006). The three main targets within the recreational fishery include the Siberian taimen (*Hucho taimen*, Pallas 1773), the sharp‐snouted lenok (*Brachymystax lenok*, Pallas 1773), and the Baikal grayling (*Thymallus baicalensis*, Dybowski 1874). Two additional species, the blunt‐snouted lenok (*B. tumensis,*Mori 1930) and the Hovsgol grayling (*T. nigrescens*, Dorogostaisky 1923), are also commonly caught and killed and are thus also suffering a similar fate across their more restricted distributions in Mongolia including the Onon River (Amur Basin) and Lake Hovsgol (Selenge Basin), respectively.

Phylogeographic research on these salmonid species has revealed population genetic structure across various geographic scales Kuang, Tong, Xu, Sun, and Yin, (2010); however, there are little or no detailed data existing for the threatened populations across the Selenge, the upper Yenisei and the upper Amur river basins in Mongolian territory. While the historical distribution of *H. taimen* encompasses most of northern Eurasia, only two major phylogeographic groups, displaying low allelic richness, have been identified previously (Froufe, Alekseyev, Knizhin, & Weiss, 2005; Maric et al., 2014). A past population bottleneck has been proposed as the likely cause of such low genetic diversity within the world's largest salmonid species, which has occurred prior to a relatively recent range expansion during a period of hydrological exchange between neighboring Siberian river basins (Froufe, Alekseyev, Knizhin, Alexandrino, & Weiss, 2003; Grosswald, 1998; Holčík, Hensel, Nieslanik, & Skacel, 1988). It has been hypothesized that if the population bottleneck occurred after the predicted range expansion, then multiple genetic lineages would have likely occurred, which appears to not be the case (Froufe et al., 2003). However, further detailed genetic research focused on *H. taimen* populations in the understudied Mongolian river systems may yet identify putative genetically independent lineages.

The *Brachymystax* genus is made up of three recognized species with two of these residing in Mongolia including *B. lenok*(sharp‐snouted lenok), the most widely distributed and commonly captured species (Kaus, 2018) and *B. tumensis*(blunt‐snouted lenok), a similar looking species which is found in more fragmented populations in the Onon River (Amur River Basin) (Bogutskaya & Naseka, 1996; Ma et al., 2007; Froufe, Alekseyev, Alexandrino, & Weiss, 2008; Xing et al., 2015). To date, both broad‐scale phylogroups and intrabasin genetic structuring have been identified for *B. lenok* between the major river basins across northern Asia/Siberia and within Chinese rivers, respectively (Froufe et al., 2008, 2003; Maric et al., 2014; Xia et al., 2006). However, the Mongolian populations of this species remain largely unstudied in detail and it is yet to be determined whether *B. lenok* displays intrabasin genetic structure outside of the highly fragmented Chinese basins. In addition, while the species status of the sympatric *B. tumensis*in Mongolia has created confusion among ichthyologists in the past, in other regions, the blunt‐snouted lenok has been demonstrated to have had a clear genetic divergence and thus been reported to have undergone a long and independent evolutionary history (Froufe et al., 2008, 2003; Shed'ko, 1996). However, due to the minimal amount of ichthyological research in Mongolia, the question has remained whether the resident blunt‐snouted lenok populations are in fact *B. tumensis*, an intraspecific form of *B. lenok*(as has been previously accepted), or a different species altogether. To date, only one lenok species (*B. lenok*) has been recorded on the country's official fish species list, including the country's Red List of Fishes (2006). Thus, this issue should be explicitly addressed using modern genetic methods in order to confirm the status of the Mongolian *Brachymystax* species and populations.

For the *Thymallus* genus, multiple lineages have been described throughout Eurasian with a number of distinct species found to display a fixed genetic divergence within a restricted distribution (Antonov, 2004; Froufe et al., 2003; Froufe, Alekseyev, Knizhin, & Weiss, 2005; Knizhin, Antonov, Safronov, & Weiss, 2007; Knizhin, Weiss, Bogdanov, Kopun, & Muzalevskaya, 2008; Knizhin & Weiss, 2009; Slynko, Mendsaykhan, & Kas'anov, 2010; Weiss, Knizhin, Romanov, & Kopun, 2007). This genetic divergence has been attributed to several characteristic traits of the genus including strong natal homing tendencies and poor dispersal abilities (Froufe, Alekseyev et al., 2005; Koskinen, Knizhin, Primmer, Schlötterer, & Weiss, 2002; Weiss, Persat, Eppe, Schlötterer, & Uiblein, 2002). In Mongolia, there are five *Thymallus* species currently listed, with *T. baicalensis* being recently confirmed as the species inhabiting the Selenge River Basin (Weiss et al., 2007) after it was previously thought to be *T. arcticus*(Pallas, 1776). However, it remains unclear whether any further *Thymallus* species reside in the expansive Selenge River system as has been suggested (Kottelat, 2006), or whether there is clear genetic sub‐structuring displayed by this species that would need to be considered in more comprehensive fisheries management plans. While the status of the Mongolian grayling (*T. brevirostris,*Kessler, 1879), the Amur grayling (*T. grubii,* Dybowski, 1869), and the upper Yenisei grayling (*T. svetovidovi*, Knizhin & Weiss, 2009) is clear, further clarification is required to determine whether the Hovsgol grayling (*T. nigrescens*, Dorogostaisky, 1923) from Lake Hovsgol in the central north of Mongolia represents a unique and independent species or not, as it has been treated as a separate species by some authors (Berg, 1962; Bogutskaya & Naseka, 2004; Pivnička & Hensel, 1978), but not by others (Froufe, Alekseyev et al., 2005; Knizhin, Weiss, & Sušnik, 2006; Koskinen et al., 2002).

In order to clarify the status of the aforementioned species, delineate their ESUs and identify genetically diverse or differentiated intraspecific populations that should be conservation priorities, a combination of mtDNA sequencing and microsatellite marker analyses was conducted on sampled individuals from each species across their entire Mongolian distributions including the upper Yenisei, the Selenge, and upper Amur river basins. It is hypothesized that the unfragmented river basins in Mongolia, which are unique for such large, boreal systems in the world, have allowed for unobstructed connectivity and thus unrestricted movement and intergenerational gene flow over large spatial scales, which has ultimately resulted in the genetic structure of these species being primarily segregated between river basins with minimal differentiation existing within each river basin. Our aim was to also shed light on the phylogenetic relationships and species status of *B. tumensis*and *T. nigrescens* using genetic techniques. This research can provide a more detailed understanding of the genetic structure of these threatened salmonid species in Mongolia, while providing authorities with an improved ecological understanding in order to develop more comprehensive management strategies and better protect rivers holding genetically valuable populations that can help to safeguard the evolutionary potential and adaptability of these species for future climatic challenges.

## 2 MATERIALS AND METHODS

### 2.1 Study area

**Figure 1:**
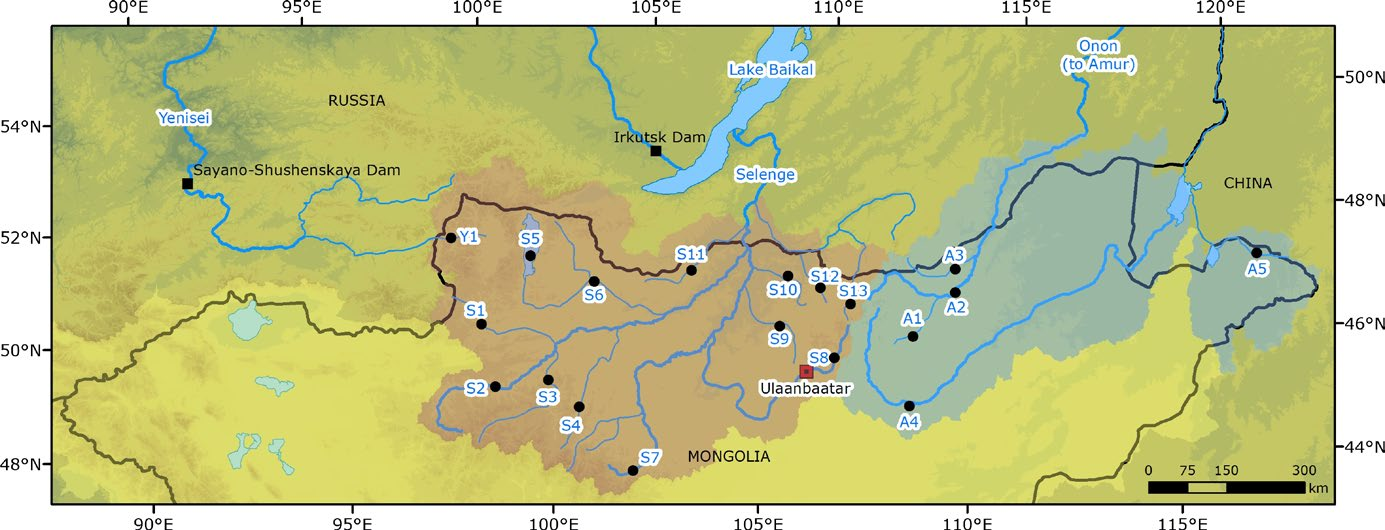
Displays the sample locations across northern Mongolia. The major river basins shown include the Yenisei/Selenge River Basin (Arctic Ocean drainage in red shading), the Amur River Basin (Pacific drainage in blue shading), and the Central Asian basin (yellow shading). Only a very upper tributary of the Yenisei River (Shishged River), which is downstream of Lake Baikal, is located in Mongolian territory and is labeled as Y1. *Hucho taimen*samples were collected from sites Y1, S1, S3, S6, S10, A2, A3, and A5; *Brachymystax lenok* samples were collected from all sample points; *B. tumensis*were sampled from A2 ‐ A4; *Thymallus baicalensis*were samples from S1–S12 (excluding S5), and *T. nigrescens*was sampled from S5 only

Mongolia contains the most upstream regions of two major Eurasian drainages (Figure 1). The Selenge River Basin, with a catchment that covers most of northcentral Mongolia, flows north into Siberia and forms the main inflow to Lake Baikal. From Lake Baikal, water continues via the Angara River into the Yenisei River which ultimately discharges into the Arctic Ocean. The Shishged River is a major tributary of the upper Yenisei basin and flows approximately 100 km from its source to the Mongolian–Russian border. The second major river basin includes upper tributaries of the Amur drainage in the northeast of the country, where the Onon and Kherlen rivers flow in an easterly direction from the Khentii Mountains into Russia and China, respectively. Although currently disjunct, there is evidence for large‐scale paleohydrological exchange between the Yenisei/Selenge and Amur river basins via the Lena River Basin during the late to post Pleistocene period. This previous connection appears to have had a predominant effect on the ichthyofaunal diversity and distribution throughout the region (Froufe et al., 2003; Grosswald, 1998, 1999; Koskinen et al., 2002)

### 2.2 Field sampling

The current sampling design was intended to capture the complete genetic diversity of the investigated species from across their entire Mongolian distributions. Fish were sampled from 19 rivers within the Yenisei, Selenge, and Amur basins in 2011 and 2012 (Table 1, Figure 1). Rivers were selected due to the reported species present, the isolation by river distance, and river accessibility. Sample sites were typically in the upper reaches, but due to the low abundances of certain species in a number of rivers, it was necessary to cover tens of kilometers in order to collect sufficient numbers of samples. We collected fin clips from a total of 127 *H. taimen* from seven rivers, 371 *B. lenok* from 18 rivers, and 274 *T. baicalensis* from 11 rivers. We also collected samples of *B tumensis* from the Amur basin (12 individuals from 3 rivers) and *T. nigrescens* from Lake Hovsgol (15 individuals). Fish were caught using backpack electrofishing units (Hans Grassel GmbH, Germany; Type ELT 60) and angling by researchers and international fishing guides, with all of these individuals being released alive. Several samples were also collected from fish caught by local recreational anglers. Fin clips were placed into 96% ethanol for transportation and storage until analysis at the Helmholtz Centre for Environmental Research (UFZ) in Halle (Saale), Germany.

**Table 1:** Genetic diversity of three salmonid genera sampled from the Yenisei, Selenge, and Amur rivers basins in Mongolia in 2011 and 2012

Sample site information			Microsatellites					mtDNA		Evolutionarily significant units		Priority rivers
Basin	River	Pop. ID	*n*	A	A_R_	H_e_	*F*_IS_	*n*	Haplotype No.	Microsatellite clusters	mtDNA groups	Contribute ^*^/exclusive haplotypes^**^
*Hucho taimen*												
Yenisei	Shishged	Y1	6	2.6	2.03	0.333	0.000	6	H1, H6^*^, H7^*^	1	1	^**^
Selenge	Delgermoron	S1	12	4.0	2.16	0.337	‐0.019	3	H1	1	1	^*^
	Chuluut	S3	4	2.1	1.93	0.269	0.072	2	H1	1	1	
	Eg‐Urr	S6	8	3.1	2.12	0.343	‐0.016	2	H1	1	1	^*^
	Eroo	S10	45	5.3	2.01	0.322	**0.053**	8	H1	1	1	
Amur	Onon	A2	44	6.3	2.49	0.424	**0.077**	7	H3/4/5	2	2	^*^
	Balj	A3	4	2.3	2.36	0.361	**0.215**	‐	‐	2	‐	
	Khalkhin	A5	4	2.7	2.71	0.492	0.153	3	H3/4/5	2	2	^*^
*Brachymystax*species including sharp‐snouted lenok (*B. lenok*) and blunt‐snouted lenok (*B*. *tumensis* *;* BT.)												
Yenisei	Shishged	Y1	9	2.1	2.13	0.338	**0.342**	5	H15	2b	2	^*^
Selenge	Delgermoron	S1	16	5.9	4.65	0.634	‐0.008	3	H16, H20^*^	1	1	^**^
	Ider	S2	17	5.4	4.38	0.598	‐0.049	‐	‐	1	‐	
	Chuluut	S3	20	5.6	4.43	0.603	**0.073**	2	H16, H21^*^	1	1	^**^
	Humen	S4	6	4.1	4.89	0.674	0.038	‐	‐	1	‐	^*^
	Hovsgol	S5	11	5.0	4.65	0.606	0.066	‐	‐	1	‐	
	Eg‐Urr	S6	24	6.3	4.66	0.614	0.000	3	H16	1	1	
	Orkhon	S7	45	6.3	4.37	0.622	**0.050**	1	H16	1	1	^*^
	Tuul	S8	13	4.4	4.31	0.576	‐0.007	‐	‐	1	‐	^*^
	Kharaa	S9	61	6.4	4.28	0.593	0.024	3	H16	1	1	
	Eroo	S10	38	6.0	4.14	0.538	‐0.002	3	H16	1	1	
	Zelter	S11	13	5.3	4.50	0.566	‐0.132	‐	‐	1	‐	
	Huder	S12	31	6.8	4.40	0.576	‐0.007	‐	‐	1	‐	
	Minj	S13	11	3.9	3.78	0.557	**0.195**	1	H16	1	1	
Amur	Barch	A1	13	6.0	4.73	0.646	**0.145**	‐	‐	2c	‐	
	Onon	A2	11	6.0	4.76	0.672	**0.102**	2	H15	2c	2	^*^
	Onon	A2‐BT	10	4.5	4.01	0.540	**0.173**	10	H15, H22^*^, H3/5/8, H23^*^	2a	2, 3	^**^
	Balj	A3	13	5.5	4.45	0.622	‐0.031	1	H15	2c	2	
	Balj	A3‐BT	1	1.5	‐	‐	‐	1	H15	2a	2	^*^
	Kherlen	A4	7	4.0	4.07	0.564	0.042	5	H15	2c	2	^*^
	Kherlen	A4‐BT	1	1.1	‐	‐	‐	‐	‐	2a	‐	^*^
	Khalkhin	A5	12	5.3	3.98	0.597	**0.101**	6	H11/12, H15	2c	2	^*^
*Thymallus*species including *T. baicalensis*and *T. nigrescens*(Lake Hovsgol)												
Selenge	Delgermoron	S1	24	10.1	6.75	0.674	‐0.093	3	H4	1	1	^*^
	Ider	S2	14	7.6	6.18	0.640	‐0.631	‐	‐	1	‐	
	Chuluut	S3	16	9.6	7.05	0.624	0.010	1	H4	1	1	
	Humen	S4	15	8.6	6.92	0.662	**0.089**	‐	‐	1	‐	
	Hovsgol	S5‐Tn	15	7.13	5.79	0.633	‐0.092	3	H4	1	1	^*^
	Eg‐Urr	S6	55	13.9	6.92	0.653	**0.043**	3	H4	1	1	
	Orkhon	S7	27	10.4	6.83	0.684	**0.081**	7	H4, H5, H6	1	1	^*^
	Tuul	S8	10	6.8	6.52	0.682	‐0.055	‐	‐	1	‐	^*^
	Kharaa	S9	24	9.4	6.05	0.607	‐0.020	4	H4, H6	1	1	
	Eroo	S10	54	14.0	6.84	0.641	**0.015**	2	H4	1	1	
	Zelter	S11	21	10.3	7.05	0.707	‐0.010	‐	‐	1	‐	^*^
	Huder	S12	14	8.1	6.69	0.699	0.009	‐	‐	1	‐	^*^

Note

The table lists the basin and river where individuals were sampled, population identification code, sample size (*n*), mean number of alleles (A), allelic richness (A_R_) (with a rarefaction sample size of 4 for *Hucho*, 6 for *Brachymystax,* and 9 for *Thymallus*), expected heterozygosity (*H*_e_), and inbreeding coefficient (*F*_IS_) where bold *F*_IS_ values indicate significance. For mtDNA data, sample size (*n*) is shown along with the haplotypes identified, where those haplotypes with an asterisk (^*^) are new. The evolutionarily significant units (ESUs) as identified for each genus according to the Contrib software (*) microsatellites and mtDNA results are shown along with the priority rivers which hold fish that displayed an above average genetic diversity or differentiation as well as those populations that exhibited new haplotypes for the species or exclusive haplotypes for Mongolian populations (designated by **).

### 2.3 Genotyping

DNA was extracted from fin clips using the DNeasy Blood and Tissue kits (QIAGEN, Hilden, Germany) following the manufacturer's instructions. We sequenced the control region (“D‐loop”) of mitochondrial DNA from a total of 31 *H. taimen* that were sampled from across seven rivers, a total of 35 *B. lenok* from across 12 rivers, and a total of 11 *B. tumensis* from two rivers, as well as a total of 20 *T. baicalensis* from across six rivers and three *T. nigrescens* from Lake Hovsgol using primers LRBT‐25 and LRBT‐1195 (Uiblein, Jagsch, Honsig‐Erlenburg, & Weiss, 2001). Details of the sequencing reaction are given in the Supplementary Material.

All *H. taimen*, *Brachymystax,* and *Thymallus* samples were genotyped at eleven, eight, and eight microsatellite loci, respectively (Supporting information Table S1). A small number of loci produced multiple bands which could be consistently scored as independent loci, one in *Brachymystax* (BleTri4) and two in *H. taimen* (BleTri4 and BleTet6). We used a PCR protocol with CAG/M13R‐tagged forward primers and GTTT‐“pigtailed” reverse primers following Schuelke (2000). Primer sequences and details of the PCR protocol are given in the Supplementary Material.

### 2.4 Data analysis

**Figure 2:**
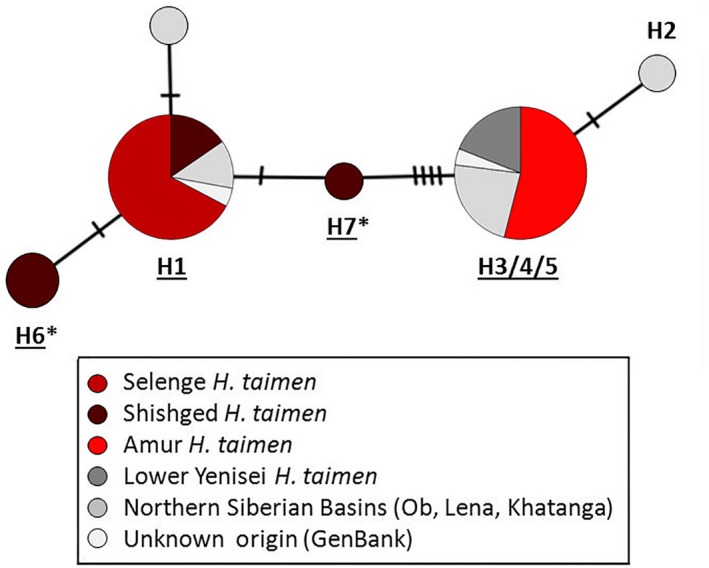
Mitochondrial DNA haplotype network for *Hucho taimen* sampled from Mongolia and from Froufe, Alekseyev et al. (2005), with haplotypes detected in this study labeled using existing haplotype names (Froufe, Alekseyev et al., 2005, Figure 2c). Haplotypes found in this study are underlines, while new haplotypes for the species are denoted with an asterisk (*). Note that some of the previously identified haplotypes (Froufe et al., 2003; Froufe, Alekseyev et al., 2005) collapsed into a single haplotype because the total alignment was shorter

Following preliminary analysis, we combined individuals from adjacent collection sites within a specific river when sample sizes were particularly low; thus, we refer to species “populations” even if sample groups represented considerable parts of the same river. Full analyses were then carried out separately for each of the three salmonid genera collected across Mongolia. Mitochondrial DNA data and additional sequences acquired from GenBank (for *Brachymystax* spp. and *H. taimen*) were aligned using Geneious® Pro 5.6.7 (Kearse et al., 2012) and the build‐in multiple alignment option. Haplotype networks were obtained by using a median‐joining algorithm (Bandelt, Forster, & Rohl, 1999) implemented in PopART v1.7.2 (http://popart.otago.ac.nz). For *H. taimen* and *Brachymystax*spp., haplotypes were labeled following Froufe, Alekseyev et al. (2005); Figure 2c) and Froufe et al. (2008; Figure 5), respectively, using new names as necessary. In the analysis of *Brachymystax,* we also included GenBank sequences of *B. tsinglingensis* from China (Liu, Li, Lui, Zou, & Wei, 2012; Xing et al., 2015).

**Figure 3:**
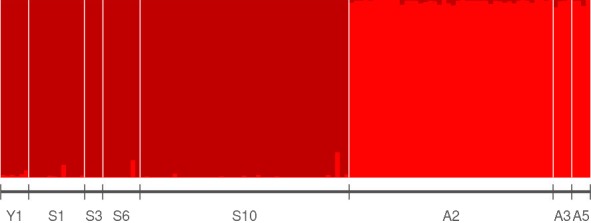
Bayesian cluster analysis with STRUCTURE for the microsatellite data of *H. taimen* sampled in eight rivers from the Yenisei (Y1), Selenge (S1, S3, S6, S10), and Amur basins (A2, A3, A5) across Mongolia. Individual proportional membership is shown for K = 2 as two genetic clusters were identified by the Evanno plots (Figure S1). Each identified cluster was again run separately and both displayed K = 1

From the microsatellite data sets, we calculated descriptors of population genetic variation, that is, the number of alleles (A), allelic richness (A_R_), expected heterozygosity (H_e_) as well as the inbreeding coefficient (*F*_IS_) and its significance (*p*‐value) using FSTAT v2.9.3.2 (Goudet, 2001). The presence of distinct genetic clusters was assessed with STRUCTURE 2.3.4 (Pritchard, Stephens, & Donnelly, 2000) where a burn‐in period of 100,000 was used and 1,000,000 Markov Chain Monte Carlo repetitions were performed with 10 replicates. This model‐based Bayesian approach excludes prior information on the origins of individuals. The number of clusters run was between K = 1 and K = number of populations +1. The most likely number of clusters was determined by evaluating both the likelihood of models and the ΔK method (Evanno, Regnaut, & Goudet, 2005). Independent runs were merged with CLUMPP 1.1.2 applying the Greedy algorithm and plotted with the Pophelper web application (Francis, 2016). When multiple clusters were found, we reanalyzed these clusters separately as the STRUCTURE software was sensitive to hierarchical population structure. Population differentiation was quantified with hierarchical analyses of molecular variance (AMOVA) conducted in GeneAlEx 6.5 (Peakall & Smouse, 2006, 2012). We tested for isolation by distance within basins, that is, a correlation between genetic differentiation and distance along the river with Mantel tests in R (R Core Team, 2013). River distance was estimated between hydrologically connected sites by tracing the main river channel and using the measuring ruler “*path*” in Google Earth 7.1.7.2600 (Google Inc., 2016). River distances ranged from 465 to 4,994 km (mean 1,637 km) in the Yenisei/Selenge basin and 256 to 3,245 km (mean 1,977 km) in the Amur basin.

Populations were assigned to separate ESU's when the combined evidence of mitochondrial and nuclear genetic data indicated genetic isolation. Genetic isolation was recognized when there was clear haplotype separation according to the mDNA data, along with distinct clustering of the concerned population/s as identified using the Evanno method for the nuclear data. Then to further identify rivers containing individuals that have an above average genetic diversity and/or were genetically distinct within each ESU, the nuclear data were used along with the Contrib Software (Petit et al., 1998). Calculations to identify priority populations or priority rivers were based on allelic richness thus correcting for unequal sample sizes. The contribution of populations to total species diversity was partitioned into two components: the diversity of individuals within that river and their differentiation from other rivers. Although genetic diversity/differentiation are common metrics used to identify priority populations within a species distribution, it is not the only definition that has been used in conservation biology studies of threatened species, with population viability, highest risk and/or greatest ecological consequences following extinction having also been used as criteria to prioritize conservation resources (Allendorf et al., 2002). In any case, within the scope of the current research, only genetic metrics have been used to delineate priority populations within the different salmonid species' ESUs. However, while this detailed genetic information has provided initial conservation priorities, ultimately these data can later be combined with ecological and demographic population assessments to further enhance salmonid species' management strategies in Mongolia.

## 3 RESULTS

### 3.1 *H. taimen* mitochondrial and nuclear markers

Four mitochondrial haplotypes were identified within the sampled *H. taimen*(Table 1, Figure 2). These could be assembled into two main groups, separated by four mutations. The first group included Selenge and Shishged individuals (identified by two different shades of darker red), while the second group was made up of Amur *H. taimen* (lighter red). All Selenge individuals grouped into a single haplotype (H1), while only some Shishged *H. taimen* were contained within this haplotype (H1). The remaining Shishged individuals displayed two additional and exclusive haplotypes (denoted with an asterisk) H6* and H7*. *H. taimen*sampled from the three Amur Basin rivers were all contained in previously documented haplotype H3/4/5.

*Hucho taimen* nuclear microsatellites indicated that the mean allelic richness (A_R_) was 2.23 (*SD*: 0.27) with a range from 1.93 in the Chuluut to 2.71 in the Khalkhin (Table 1). Mean allelic richness was higher in the Amur (mean 2.52, *SD*: 0.18) than the Selenge (2.06, *SD*: 0.10) basin, while the Shishged *H. taimen* had an A_R_ of 2.03. Three out of eight rivers that *H. taimen* were sampled, including both the Selenge and Amur, showed significant inbreeding coefficients (S6, A2, and A3). STRUCTURE analyses revealed that *H. taimen* displayed two genetic clusters, the first consisting of all sampled Selenge basin rivers and the Shishged River, while the second cluster included the Onon and Khalkhin rivers, respectively (Figures 3, S1a). No further genetic structure was evident when each basin cluster was analyzed again separately (Figure S1b and c). These clusters were also supported by the principal component analysis (PCoA), where axis one explained 31.7% of the variation and axis two explained 7.7% of the variation (Figure S6). The AMOVA for *H. taimen* indicated that 29% of the genetic variance was partitioned among basins, 1% among rivers within basins, and the rest residing within rivers (Table S5a). Separate analyses for the Selenge (incl. Shishged) and Amur basins revealed *F*_ST_ = 0.027 (*p* = 0.001) and *F*_ST_ = 0.052 (*p* = 0.008), respectively (Table S5b and c). The overall *F*_ST_ value for *H. taimen* was 0.302.

**Figure 4:**
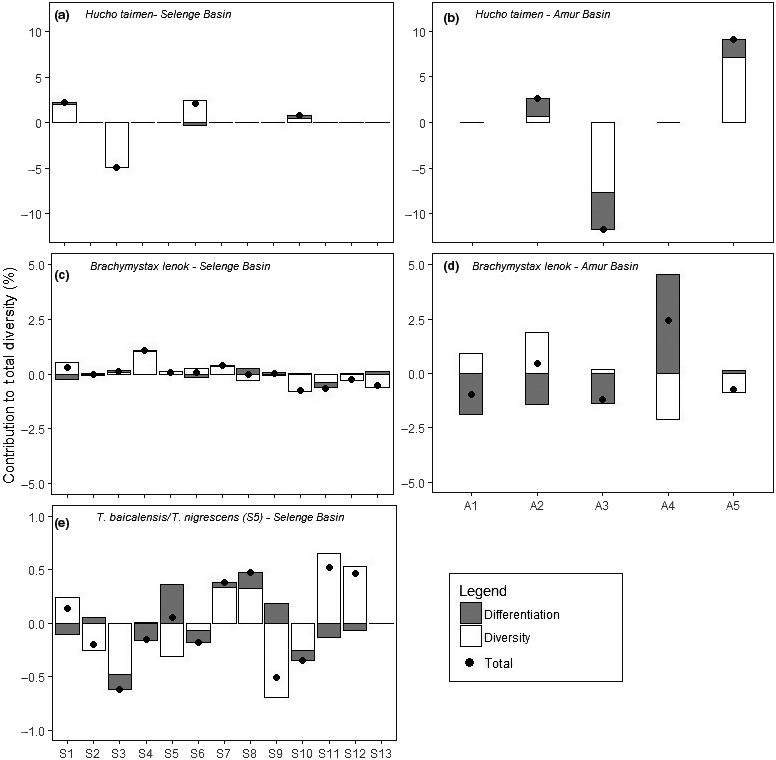
Displays the contribution of each population to total diversity based on allelic richness, thus correcting for unequal sample sizes. Shown are populations of *H. taimen*(top)*, B. lenok* (excluding *B. tumensis*) (center) and *T. baicalensis* (bottom, including *T. nigrescens* in S5) from the Selenge (left) and Amur (right) river basins, Mongolia. The black dots indicate total genetic diversity partitioned into the contribution of genetic diversity within the population (white bar) and contribution of genetic differentiation of the population (gray bar). Note that the Shishged River (Yenisei basin) population was not included in the analysis due to their large genetic differentiation from the other Selenge River Basin groups sampled

Priority rivers within each species' ESU were identified using the contrib analysis software as the populations that recorded the greatest contribution percentage to the total genetic diversity based on allelic richness. This metric allowed populations of unequal sizes to be compared without bias and the total genetic diversity to be partitioned into the contribution of genetic diversity within the population and the contribution of genetic differentiation of a population within an ESU (Figure 4, Table 1). For *H. taimen* from three rivers in the Selenge (Shishged, Delgermoron, and Eg‐Uur) and two in the Amur (Onon and Khalkhin), we identified increased genetic diversity and thus were recognized as priority rivers (Figure 4a and b).

### 3.2 *Brachymystax* spp. mitochondrial and nuclear markers

**Figure 5:**
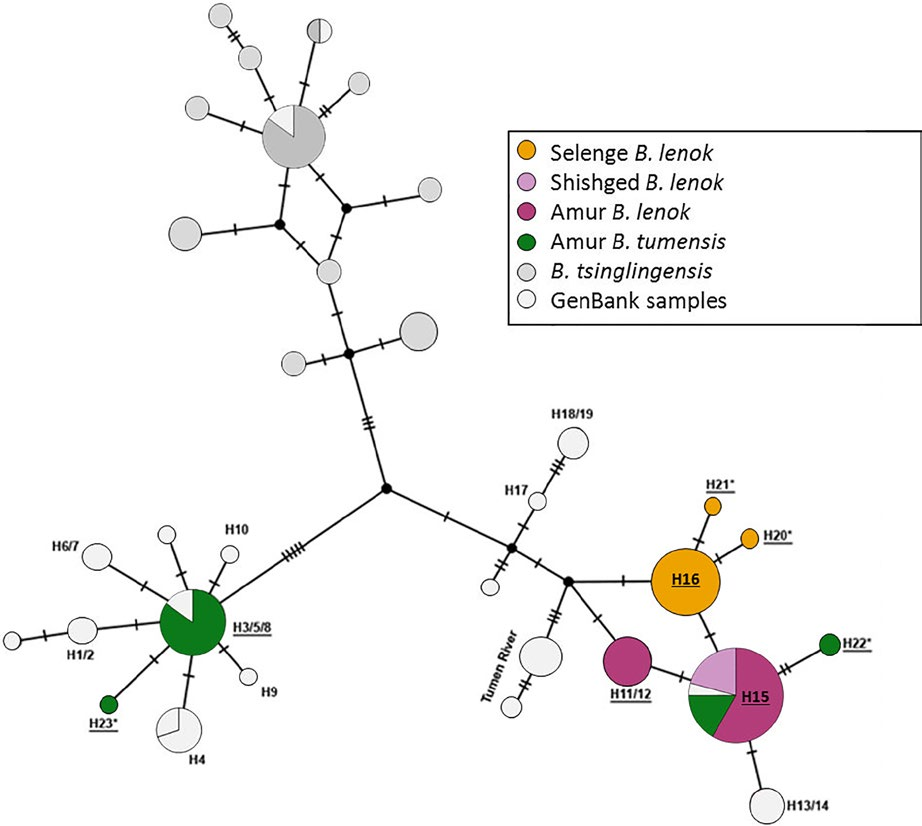
Mitochondrial DNA haplotype network of *Brachymystax* species, that is, sharp‐snouted lenok (*B. lenok*), blunt‐snouted lenok (B. *tumensis*) sampled from Mongolia, and *B. tsinlingensis* from GenBank. Haplotypes found in this study are underlined, haplotype names used in Froufe et al. (2008, 2003) were maintained, and new haplotypes are denoted with an asterisk (*). Note that some of the previously identified haplotypes (Froufe et al., 2008, 2003) collapsed into a single haplotype because the total alignment was shorter

A total of eight mtDNA haplotypes were identified within the sampled *Brachymystax* individuals (Table 1, Figure 5). For *B. lenok,* a clear group of three haplotypes (H16, H20*, and H21*) was observed and exclusively associated with the Selenge River Basin. *B. lenok* sampled from the Amur and Shishged basins displayed a further three haplotypes (H11/12, H15, and H22*), which together formed a second, separate group. Three of the *B. lenok* haplotypes had been found previously, while the other three haplotypes (denoted with an asterisk) were closely related but new for the species. Haplotypes identified in *B. tumensis* belonged to two highly divergent groups. Some individuals contained two haplotypes from a *B. tumensis*‐specific group (H3/5/8 and H23*), one of which was new. However, many of the *B. tumensis*samples displayed the common *B. lenok* haplotype (H15), while some individuals also made up the H22* haplotype.

**Figure 6:**
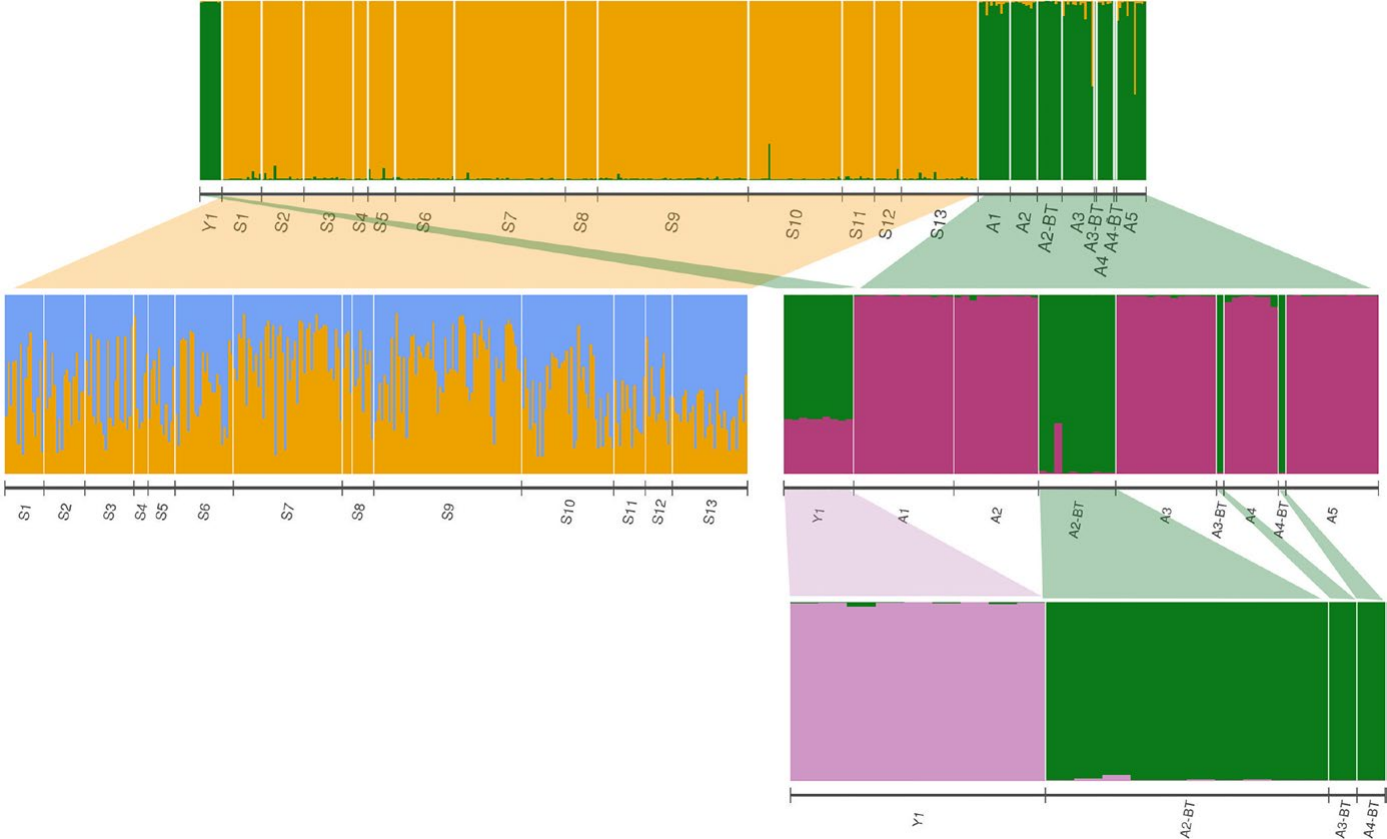
STRUCTURE analyses of the microsatellite data for the Brachymystax genus (including both *B. lenok* and *B. tumensis*) collected from 19 rivers across the Yenisei (Y1), Selenge (S1–S13), and Amur river basins (A1–A5) in Mongolia. When all samples were included in the analysis, two genetic clusters were identified according to the Evanno plots (top; Figure S2a). These two clusters were further analyzed separately (second row), with the results from the “orange cluster” (Selenge basin) yielding no further genetic structure (Figure S2b), while within the “green cluster,” additional sub‐structuring was identified (Figure S2c). Upon further analysis, no substructure of the “purple cluster” was identified (results not shown). However, within the “green cluster,” two genetically distinct populations were clearly displayed (Figure S2d): *B. lenok* from the Shishged River (pink cluster) and *B. tumensis* from the Onon and Kherlen rivers (green cluster)

For nuclear microsatellites, low A_R_ was found in the Shishged (Y1: A_R_ = 2.13) population, whereas much higher values were evident in the Selenge (mean 4.42, *SD*: 0.28) and Amur River basin populations (mean 4.40, *SD*: 0.36). In each river basin, there was at least one population that had significant *F*_IS_ values indicating deviation from Hardy–Weinberg equilibrium (HWE). *B. tumensis* in the Onon River had similar values of genetic variation as *B. lenok* with significant *F*_IS_ values. STRUCTURE analyses of *Brachymystax* samples (including both *B. lenok*and *B. tumensis*) revealed a total of four genetic clusters (Figures 6, S2, S3, S4). The first analysis displayed two distinct genetic clusters within the genus, which represented the separation of individuals in the Selenge (orange) from the Amur and Shishged (green, Figure 6, first structure level, Figure S2a). Notably, the sympatric *B. lenok* and *B. tumensis* from the Amur clustered together rather than forming separate species clusters. The Selenge cluster (orange) displayed no further substructure in additional analyses (Figure 6, second structure level; Figure S2b). In contrast, the green cluster, which included both the Shishged and Amur, displayed additional substructure (Figure 6, purple and green groups, Figure S2c). *B. lenok* from the Amur basin (purple) formed a distinct genetic cluster, as it separated from both *B. tumensis* in the Amur and *B. lenok* from the Shishged (green), which remained clustered together. However, upon further analysis, *B. tumensis* (green) was shown to be genetically distinct from the Shishged *B. lenok* (pink, Figures 6, S2d, S3, S4). Thus, the *Brachymystax* genus comprised of four genetic clusters in Mongolia, representing *B. lenok* from the Selenge, Shishged, and Amur basins as well as the sympatric *B. tumensis*from the Amur. This structure was also corroborated both by the pairwise *F*_ST_ values (Table S3) and the PCoA (Figure S7). The AMOVA results for *B. lenok* indicated that 16% of the genetic variance was among basins, 2% among rivers within basins, and the rest residing within rivers (Table S5e) with an overall *F*_ST_ value of 0.181. Population differentiation among *B. lenok* was similar in the Amur (*F*_ST_ = 0.056) and the Selenge basin (*F*_ST_ = 0.049). Mantel tests indicated a pattern of isolation by distance for *B. lenok* in both the Selenge (*r* = 0.41, *p* = 0.004; Figure S9a) and the Amur basin (*r* = 0.76, *p* = 0.045; Figure S9b). For *B. lenok*, five priority rivers were identified in the Selenge (Shishged, Delgermoron, Humen, Orkhon, and Tuul; Figure 4c) and two in the Amur (Onon and Kherlen; Figure 4d).

### 3.3 *Thymallus* spp. mitochondrial and nuclear markers

**Figure 7:**
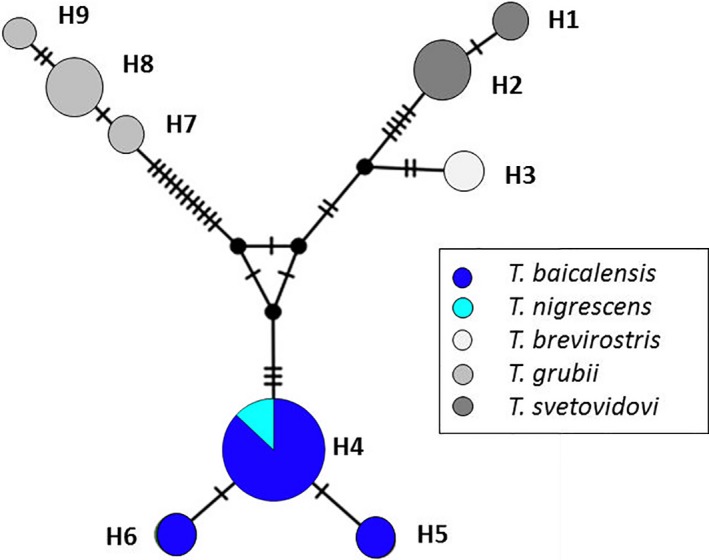
Mitochondrial DNA haplotype network for *Thymallus*species (*T. baicalensis, T. grubii, T. nigrescens, T. svetovidovi,*and *T. brevirostris*) sampled from the Shishged (Yenisei), Selenge, Amur, and Central Asia river basins in Mongolia

Nine mtDNA haplotypes were found among five *Thymallus* species. The haplotype network showed four distinct groups (Figure 7). Three of these groups were comprised of haplotypes from single species samples taken from GenBank with the *T. brevirostris* group having one haplotype (H3), *T. svetovidovi* having two haplotypes (H1‐H2), and *T. grubii* having three haplotypes (H7‐H9). The fourth group comprised of a single haplotype that included *T. baicalensis* and *T. nigrescens* with both species sharing the most common haplotype (H4), with two additional rarer haplotypes (H5 and H6) found in *T. baicalensis*.

The nuclear microsatellites results indicated that the mean A_R_ was 6.72 (*SD*: 0.31) across the Selenge basin, with *T. nigrescens*from Lake Hovsgol displaying an A_R_ of 5.79. Four *T. baicalensis*populations had significant *F*_IS_ values indicating deviation from HWE. Both the structure and PCoA analyses revealed only one genetic cluster for *T. baicalensis* and *T. nigrescens*(Figures S5 and S8). This is corroborated by very weak genetic structure amounting to only 1% of molecular variance among populations (Table S5h). The overall *F*_ST_ for *T. baicalensis* and *T. nigrescens* according to the AMOVA results was 0.014. However, *T. nigrescens* was more strongly differentiated from all other *T. baicalensis* populations as the mean pairwise differentiation among *T. baicalensis*was *F*_ST_ = 0.014 but *F*_ST_ = 0.033 between the two taxa (Table S4). *T. baicalensis*across the Selenge basin showed no significant pattern of isolation by distance (*r* = 0.22, *p* = 0.12, Figure S10). For *T. baicalensis* (including *T. nigrescens*), six rivers were considered to be priorities due to the above average genetic diversity or differentiation (Delgermoron, Orkhon, Tuul, Zelter, Huder, and Lake Hovsgol; Figure 4e). In most cases, total diversity was determined by high diversity contributions rather than differentiation contributions, in line with low within‐basin divergence.

## 4 DISCUSSION

The identification of genetically diverse or distinct intraspecific groups within a species can help to define biologically relevant units that can ultimately improve the success of conservation and management strategies long term. While biological, ecological, logistical, geographical, and administrative factors are typically considered when designing species conservation plans, it has only been relatively recently that measures of genetic diversity have also been included (Hoban et al., 2013). Such an oversight can have substantial consequences for a species, as the loss of genetic variation can reduce the evolutionary potential at both a population and species level (Barrett & Schluter, 2008; Keller & Waller, 2002; Rivers, Brummitt, Nic Lughadh, & Meagher, 2014). For three taxa of salmonids with high conservation concern in Mongolia, we found that population structure was primarily segregated between major river basins with largely matching patterns between mitochondrial and nuclear genomes. Although exact patterns were not completely concordant among species, we identified strong genetic differentiation among basins but rather weak differentiation within basins. *B. lenok* was the only species to show a clear pattern of within‐basin isolation by distance. Furthermore, patterns of diversity and differentiation allowed for the identification of conservation priority rivers across Mongolia's major basins, with the results indicating that some rivers are valuable for two or more of the sampled salmonid species making them genetic hotspots. Therefore, new management strategies need to recognize the importance of understanding and incorporating genetic diversity and differentiation patterns so a more targeted approach can be developed in an attempt to retain the maximum genetic variation across intraspecific groups and maintain the highest adaptive potential of the focus species.

### 4.1 Genetic population structure and priorities for conservation of *H. taimen*

**Figure 8:**
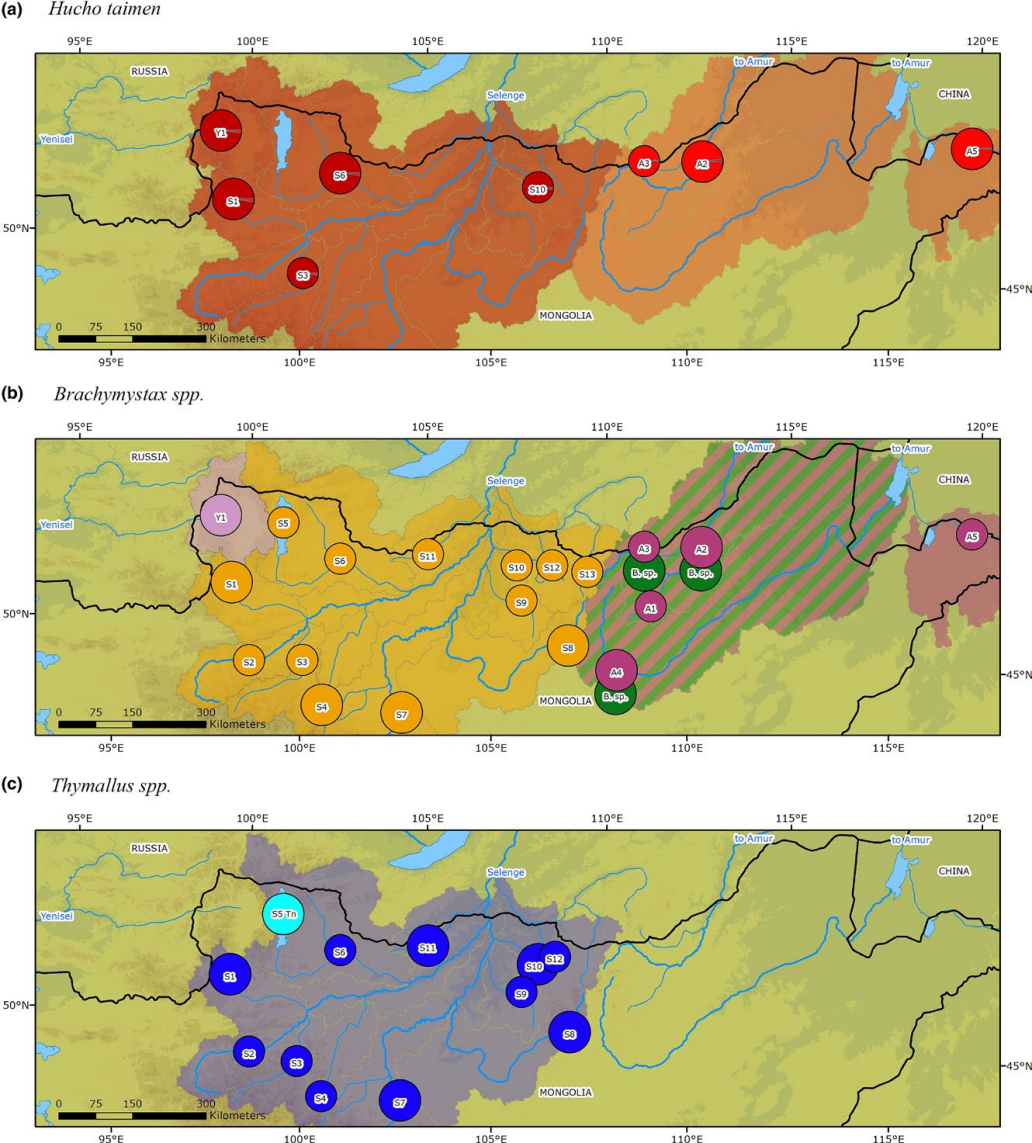
Individual species maps displaying, in different colored shading, the evolutionarily significant units (ESUs) that have been identified for (a) *Hucho taimen,*(b) *Brachymystax*spp. (*B. lenok* and *B. tumensis*), and (c) *Thymallus* spp. (*T. baicalensis*and *T. nigrescens* – S5) within Mongolia's river systems. ESUs were defined by using both the mtDNA and microsatellites results. The priority rivers for each species are represented by a larger circles

This study has demonstrated that Mongolian *H. taimen* represents the most upstream extent of the two previously identified major phylogroups, which means that two independent ESUs need to be recognized in conservation and management efforts in the country (Figure 8a). The Selenge and Shishged ESU forms part of the western phylogroup that consists of the greater Yenisei, Khatanga, Volga, and Ob river basin *H. taimen*, while the Onon and Kherlen ESU is part of the eastern Amur phylogroup together with Lena basin *H. taimen* (Froufe, Alekseyev et al., 2005; Maric et al., 2014). Certain ecological traits such as the *H. taimen's* large body size and propensity of mature individuals to move and disperse extensive distances particularly during the spawning season (Holčík et al., 1988; Matveyev, Pronin, Samusenok, & Bronte, 1998; Jensen et al., 2009; Gilroy et al., 2010; Kaus, Büttner et al., 2016) has likely contributed to the minimal genetic structure found in this long‐lived species. Similar patterns of negligible genetic structure across large geographic scales have also been reported in other large‐bodied freshwater fishes, which are also known to move extensive distances during their lifetimes (Ferreira et al., 2017; So, Houdt, & Volckaert, 2006; Stepien, Murphy, Lohner, Sepulveda‐Villet, & Haponski, 2009). Resident *H. taimen* in the Shishged River coupled with individuals from the Delgermoron, Eg‐Uur, Onon, and Khalkhin rivers collectively represent the most genetically diverse populations within the two ESUs identified in Mongolia (Figure 8a). Incidentally, these rivers also are known to hold some of the last remaining robust *H. taimen*populations in Mongolia and thus their protection will be critical, for conserving not only their genetic diversity, but for the persistence of the entire species.

### 4.2 Genetic population structure and conservation priorities for *Brachymystax* spp.

For the two *Brachymystax* species residing in Mongolian rivers, the genetic analysis revealed a total of four distinct ESUs (Figure 8b). We identified three allopatric ESUs belonging to *B. lenok* from the Selenge, the Shishged and Amur basins, and confirmed *B. tumensis* from the Amur as a separate, sympatric species and distinct ESU. The Selenge ESU formed an exclusive genetic group based on mtDNA and nuclear markers, which would likely extend as far as Lake Baikal downstream considering the findings of Froufe et al. (2008). Such genetic divergence can be attributed to the reported prior isolation of the Selenge basin and Lake Baikal from the Yenisei and Amur basins approximately half a million years ago (Mats et al., 2001 2001). This unique *B. lenok* phylogroup is genetically distinct and thus highly valuable in the context of genetic conservation for the species. Incidentally, this group has also been the focus of increased scientific research on *B. lenok* including studies on their feeding ecology (Olson, Jensen, & Hrabik, 2016), thermal tolerances (Hartman & Jensen, 2016), lotic and lentic growth comparisons (Tsogtsaikhan et al., 2017), and seasonal movements (Kaus, Büttner, Karthe, Schäffer, & Borchardt, 2017).

The other two *B. lenok* ESUs in the Shishged River and Amur basin displayed no distinction at the mtDNA level but were genetically separated from each other according to nuclear microsatellite markers. Thus together with their geographic isolation, their status as separate ESUs was justified. The shared mtDNA haplotype that was found between these ESUs highlighted the relatively recent divergence of these populations, while supporting the hypothesis of the late Pleistocene hydrological connectivity between the Amur and Yenisei basins via the Lena River (Froufe et al., 2008, 2003; Grosswald, 1998). However, despite this shared haplotype, most of the genetic differentiation for *B. lenok* was distributed among basins (Froufe et al., 2008; Liu, Kunag, Tong, & Yin, 2011; Xia et al., 2006), which indicated large‐scale, intrabasin gene flow within these vast, unfragmented river systems. However, *B. lenok* was the only species to demonstrate isolation by distance within both river basins, which is in line with the expectation of a reduced dispersal ability compared to the larger sized *H. taimen* (Yoon et al., 2014; Gilroy et al., 2010; Kaus, Büttner et al., 2016; Kaus et al., 2017).

The current results additionally demonstrate that the sympatric populations of *B. lenok* and *B. tumensis* from the Amur basin are genetically highly divergent. Thus, *B. tumensis* represents a fourth ESU for the *Brachymystax*genus in Mongolia. While natural hybrids have been identified between these two species in regions of sympatry, there has been no evidence of shared haplotypes ever occurring (Froufe et al., 2008; Ma & Jiang, 2007). However, in contrast to these previous studies, strong evidence was found for nuclear introgression from *B*. *lenok* into *B*. *tumensis*. Although this indicates incomplete reproductive isolation, there was only one first generation hybrid identified in the low number of samples collected, thus suggesting there is, at least, a certain level of mitochondrial introgression still occurring between these species in this region of sympatry. This rarity of mixed ancestry in general indicates that hybridization is infrequent or was largely an ancient event. Haplotype sharing could principally be also due to shared ancestral polymorphism, but hybridization appears more likely to be the case, as this is the first such observation reported in these species. Hybridization between congeneric fish is common especially after secondary contact and molecular markers are highly suited to test specific hypotheses (Hänfling, Bolton, Harley, & Carvalho, 2005). However, the present data set is too limited to allow for more detailed conclusions. In any case, *B. tumensis* should be formally recognized on an updated species list of Mongolian fishes and be further afforded comprehensive protection to prevent this already rare species with highly fragmented populations from declining further or becoming regionally extinct. Moreover, our results rekindle the discussion regarding the species status of *B. tumensis*and its correct taxonomic classification, as the currently used scientific name (*B. tumensis*) as well as *B. savinovi*, which has also been incorrectly applied to this species in Mongolia previously, have since been revealed to be misidentifications of *B. lenok* in the Tumen River (China/North Korea) and Lake Markakol (Kazakhstan), respectively (Alekseev & Osinov, 2006; Ma et al., 2007). Consequently, these names are regarded as invalid and thus a new scientific name for *B. tumensis* should be assigned (Froufe et al., 2008; Ma et al., 2007).

Priority rivers for conservation within the *Brachymystax* genus not only include the distinct *B. lenok* ESU from the Shishged and the *B. tumensis*ESU from the Amur, but also an additional six *B. lenok* populations that were identified within the Selenge and Amur basins (Figure 8b). With both of these basins being extensively sampled, including all major rivers, it is obvious that the overall genetic differentiation of this species in Mongolia was low. It was only the Kherlen population that displayed substantial genetic differentiation compared to the other populations and thus the Kherlen River should be earmarked for additional protection measures, particularly as the upper reaches of this river is a popular fishing destination for a growing number of Ulaanbaatar residents due to its close proximity and easy access from the capital.

### 4.3 Genetic population structure and conservation priorities for *Thymallus* spp.

In contrast to common ecological characteristics of the *Thymallus* genus such as strong natal homing tendencies and poor dispersal abilities, *T. baicalensis*in the Selenge basin has shown no evidence of genetic structuring among the sampled rivers, which is likely due to not only the comparatively smaller geographic scales but also the high hydrological connectivity that has persisted across the basin. Thus, *T. baicalensis* in Mongolia represents a single ESU including individuals from Lake Hovsgol (Figure 8c). As a result, this finding has flow on implications for the species status of *T. nigrescens* from Lake Hovsgol. While some authors have recognized it as an independent species based on both morphological and biological indices including number of gill rakers and pyloric caeca (Berg, 1962; Bogutskaya & Naseka, 2004; Pivnička & Hensel, 1978), other authorities have expressed the need for additional analyses or have already outright disregarded *T. nigrescens* as its own species (Knizhin et al., 2006; Koskinen et al., 2002; Weiss et al., 2007). The result from the current research supports the opinion that *T. nigrescens* is not genetically distinct from *T. baicalensis* and that these two putative species are in fact synonyms. The lack of genetic distinction detected by the mtDNA marker analysis suggests there is either a significant amount of contemporary gene flow between Lake Hovsgol and Selenge basin inhabitants via the Eg‐Uur River or else it has ceased only recently. The morphological differences displayed by individuals that inhabit Lake Hovsgol, including significant differences in the length–weight and age–length relationships compared to *T. baicalensis* sampled from riverine environments (Tsogtsaikhan et al., 2017), are likely due to the high ecological flexibility and phenotypic plasticity of this genus, which has previously caused confusion between intraspecific forms in Lake Baikal (i.e., black and white Baikal graylings, Knizhin et al., 2006). However, the contrib analysis and the pairwise *F*_ST_ values still indicated that individuals sampled from Lake Hovsgol, while not a separate species, displayed genetic differentiation from each other, which justifies the Lake Hovsgol population as a priority within the *T. baicalensis*ESU. Furthermore, our analysis assessed largely neutral genetic variation and does not exclude the possibility that the selection has affected ecologically important genetic variation. Therefore, the Lake Hovsgol population should still be recognized as a unique intraspecific group that should be afforded adequate conservation and protection efforts to mitigate the growing number of impacts in the region including overfishing, increased pollution, and climate change, which have been reported to be increasingly impacting this ancient lake (Ahrenstorff, Jensen, Weidel, Mendsaikhan, & Hrabik, 2012; Free, Jensen, & Mendsaikhan, 2016).

For *T. baicalensis,* only minimal differences in the genetic diversity and differentiation contributions were detected across the Selenge ESU. However, in addition to the Lake Hovsgol population, *T. baicalensis* from five other sampled rivers displayed above average genetic diversity with the Zelter and Huder, and Orkhon and Tuul, appearing to share the same proportion of genetic contribution (Figures 4e and 8c). This is likely due to the close proximity of the river confluences, and thus, a substantial amount of genetic exchange is expected to have caused this similarity. The Delgermoron was the fifth population that was identified as having an elevated genetic diversity component compared to populations from other rivers, thus also making it a priority river for the conservation of this species in Mongolia.

### 4.4 Patterns across species and implications for broader conservation strategies

While a broad understanding of population genetics is crucial for threatened species management, neutral marker patterns represent only one fundamental aspect for defining conservation objectives, and thus, a range of other biological, ecological, and economically important factors should also be considered during the development of any species' management strategy (Abell, Allan, & Lehner, 2007; Suski & Cooke, 2007; Granek et al., 2008). However, the focus of the current study was to define ESUs and priority rivers within the three genera in Mongolia based on genetic markers. Thus, our results determined that the most prominent genetic structures existed between major river basins, although there was not complete concordance among species, which is likely due in part to a number of both biological and ecological differences including natal homing tendencies, site fidelity, and species mobility. The paleogeography of these extensive river systems appears to have been the dominant influence on the genetic structure of these species, with the isolation of the Selenge/Baikal basin creating an exclusive phylogroup of *B. lenok*, as well as the genetically distinct *T. baicalensis*species. The ancient hydrological connectivity between the Yenisei and Amur has also resulted in shared genetic material between *B. lenok* populations in these currently disjunct basins. Thus, we found that the ESUs identified for both *H. taimen* and *B. lenok* included two of their major phylogeographic lineages, while the single ESU identified for *T. baicalensis*represents a large proportion of the species entire geographic distribution. Thus, Mongolia has a unique responsibility to the survival of all three of these threatened salmonid species across their extended ranges going forward.

The identification of genetically important priority rivers within each ESU can now provide a more focused approach for fisheries management and conservation efforts within Mongolia. Protection of the most genetically diverse and differentiated populations is critical, especially as all species investigated already displayed a remarkable lack of genetic structure. A preliminary conservation strategy for Mongolian salmonids may be to link priority river protection across species in order to even further maximize the resources available to authorities. Therefore, the Shishged, Delgermoron, Orkhon, Tuul, and Onon rivers represent genetic hotspots as each was deemed important for two or three of the species investigated and thus these regions should be made the focus of initial conservation efforts along with Lake Hovsgol for *T. baicalensis* and the Kherlen River for *B. lenok* (Figure 8a–c). If critical habitat can be sufficiently protected, local fish densities are likely to increase with a higher number of individuals then being able to emigrate to neighboring river systems over time (Abell et al., 2007). A key recommendation for targeting these genetically valuable populations would be to establish a network of spatially protected areas to improve their overall protection and survival. Freshwater Protected Areas (FPAs) have been successfully implemented in many countries around the world to conserve genetic diversity and aid in the preservation and recovery of threatened and exploited fish populations (Abell et al., 2007; Suski & Cooke, 2007).

## 5 CONCLUSIONS

Attaining a detailed knowledge of the genetic structure and intraspecific diversity of the main target species in Mongolia's rapidly emerging recreational fishery will help guide necessary improvements and benefit the development of more comprehensive national fisheries conservation and management strategies. This information is particularly important due to the current widespread anthropogenic pressures that continue to impact resident fish populations. Additionally with this new understanding, future translocations or introductions of genetically dissimilar individuals can be avoided and inbreeding minimized within the remaining fragmented populations (Balakirev, Romanov, Mikheev, & Ayala, 2013; Hänfling, Durka, & Brandl, 2004; Hänfling & Weetman, 2006; McDougall, Welsh, Gosselin, Anderson, & Nelson, 2017; Slynko et al., 2015). However, in order to improve the outcomes of any newly proposed protection measures, their inclusion into relevant legislation such as the Mongolian Law on Hunting (Compendium of Environment Law and Practice, Ulaanbaatar, Mongolian 2000) would be necessary for enhancing their widespread and ongoing compliance across the country.

Internationally, many of the challenges facing Mongolian salmonids are also impacting these species throughout their distributions in both Russia and China, with perturbations occurring at a far greater intensity due to higher human population densities, a more established fishing culture, lack of comprehensive fisheries management strategies and increased large‐scale infrastructure river development projects (Knizhin and Weiss (2009); Tong, Kuang, Yin, & Zhang, 2013; Zolotukhin, 2013). As a result, populations of these species have suffered from even more dramatic losses with *H. taimen* having gone locally extinct or suffered from significant declines in 39 out of the 57 river basins assessed throughout Russia (M. Skopets unpubl. data, in Hogan & Jensen, 2013), while in China *H. taimen* populations in the Heilongjiang (Amur) River have declined by 95% over the past 50 years (Tong *pers. comms*., in Hogan & Jensen, 2013). Thus, even more urgent actions are needed in both of these countries to avoid further declines and growing local and regions extinction rates. The potential to effectively transfer the current Mongolian research results and recommendations to help guide such changes is highly plausible and necessary as long as intraspecific genetic variation can be determined and new management measures effectively implemented within the existing jurisdictional framework relating to regional fisheries strategies. Closer cooperation among scientific and governmental entities at all levels, a proposal that has been made recently in Europe (Weiss, Kopun, & Sušnik Bajec, 2013), could also help to advance the management of these and other highly mobile threatened species across northern Asia. Unfortunately, the current lack of political cooperation and environmental management coordination between countries, particularly in regard to the delegation of natural resources, remains an even greater barrier to forming transnational joint fisheries management plans across the region.

## AUTHOR CONTRIBUTION

AK, DB, WD, and BH participated in the project design and coordination. AK coordinated and lead the sample collection. AK, WD, and SM carried out the laboratory work and data analysis and along with BH interpreted the results. AK wrote the manuscript. AK, SM, and DK produced the figures and maps. All authors reviewed, edited, and approved the final submitted manuscript.

## Data Availability

The authors agree to submit the complete data set obtained during the current study to GenBank upon acceptance of this manuscript in order for it to be publically available by publication.
