# Corrigendum

**DOI:** 10.1002/ece3.6081

**Published:** 2020-03-13

In “Fish conservation in the land of steppe and sky: Evolutionarily significant units of threatened salmonid species in Mongolia mirror major river basins”, which was published in issue 6, March 2019, an additional file has been included in the supporting information and a new sentence under Section 2.3 is printed below:

Accession numbers of newly generated DNA sequences are listed in SM Table 7.
