# Supplementary figures and images for: Fish conservation in the land of steppe and sky: Evolutionarily significant units of threatened salmonid species in Mongolia mirror major river basins
Source: Ecol Evol. 2019 Feb 27;9(6):3416–33. doi: 10.1002/ece3.4974 (PMC6434579; doi:10.1002/ece3.4974)

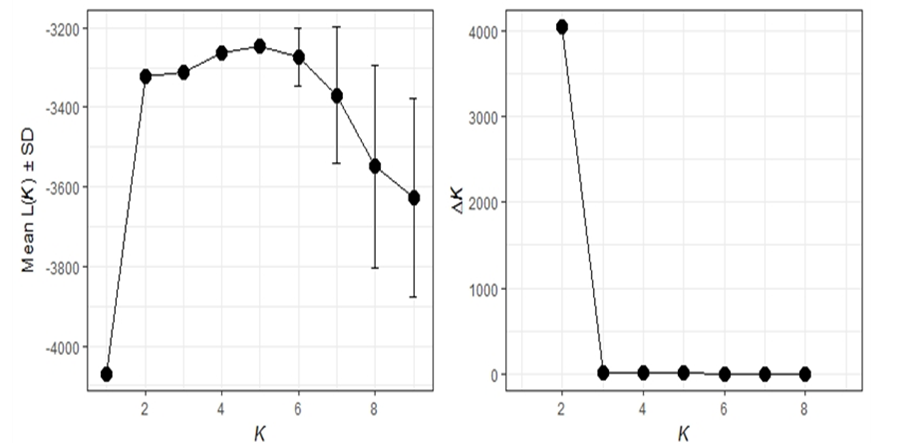

Supplement: Supplementary file 1 [file ECE3-9-3416-s001.tif]

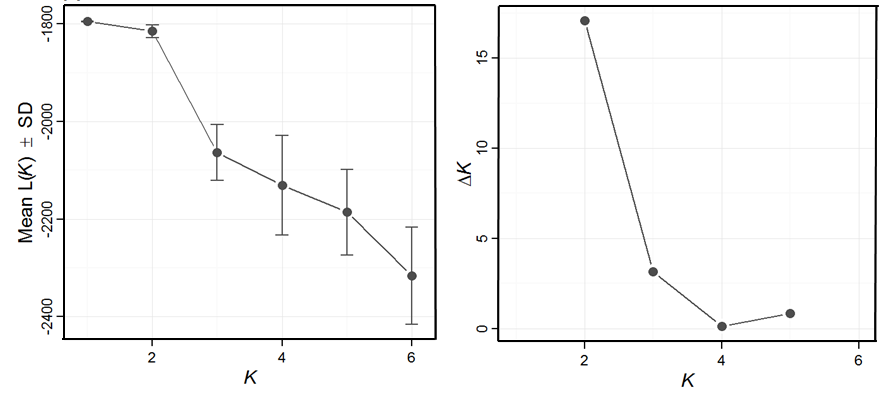

Supplement: Supplementary file 2 [file ECE3-9-3416-s002.tif]

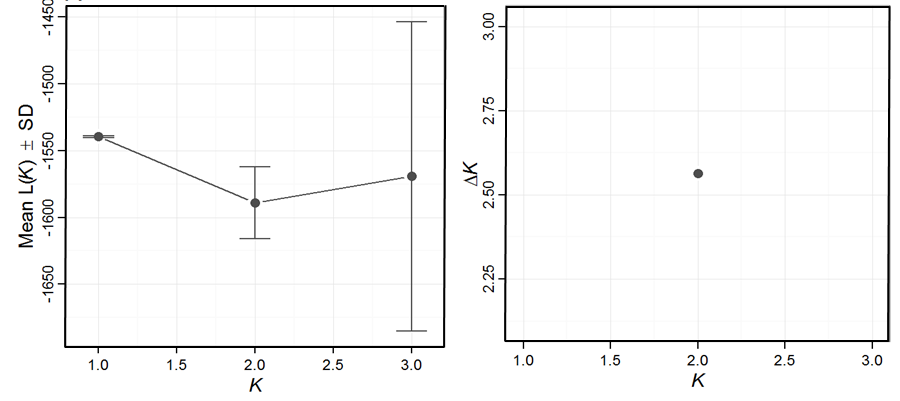

Supplement: Supplementary file 3 [file ECE3-9-3416-s003.tif]

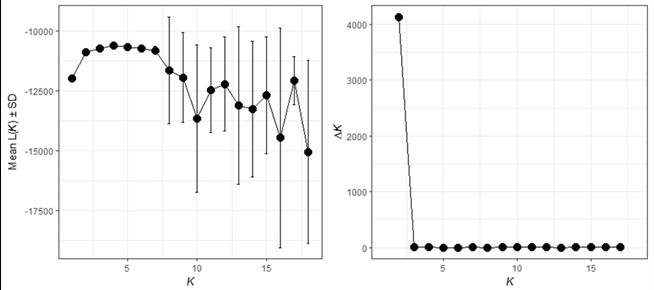

Supplement: Supplementary file 4 [file ECE3-9-3416-s004.tif]

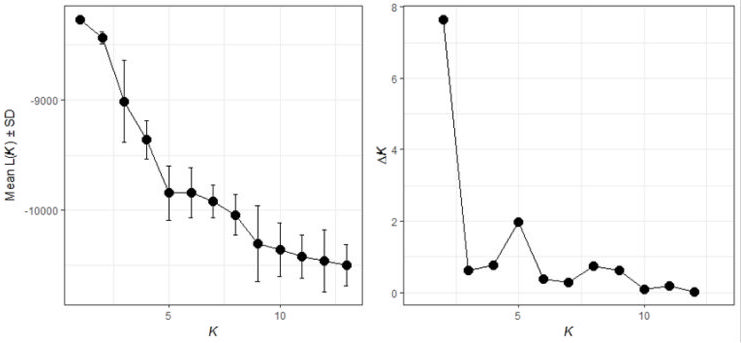

Supplement: Supplementary file 5 [file ECE3-9-3416-s005.tif]

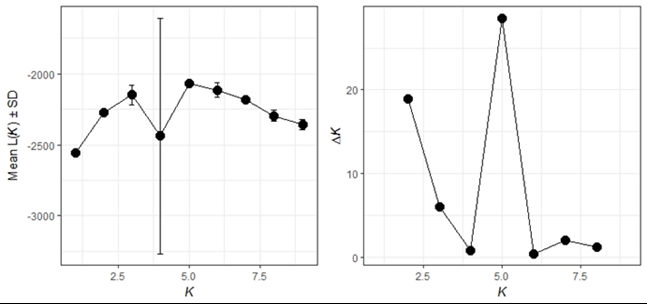

Supplement: Supplementary file 6 [file ECE3-9-3416-s006.tif]

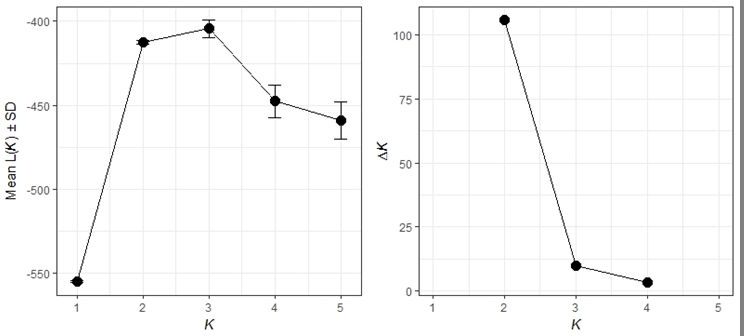

Supplement: Supplementary file 7 [file ECE3-9-3416-s007.tif]

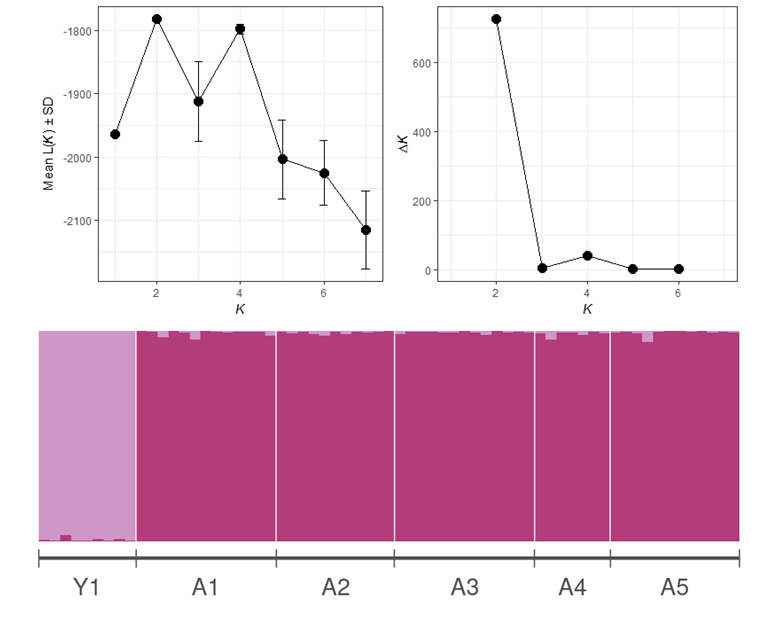

Supplement: Supplementary file 8 [file ECE3-9-3416-s008.tif]

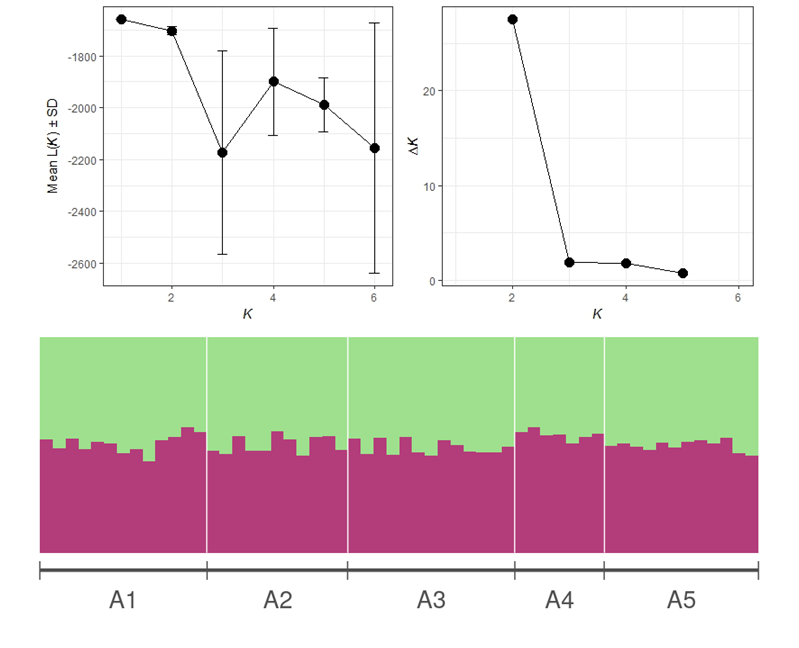

Supplement: Supplementary file 9 [file ECE3-9-3416-s009.tif]

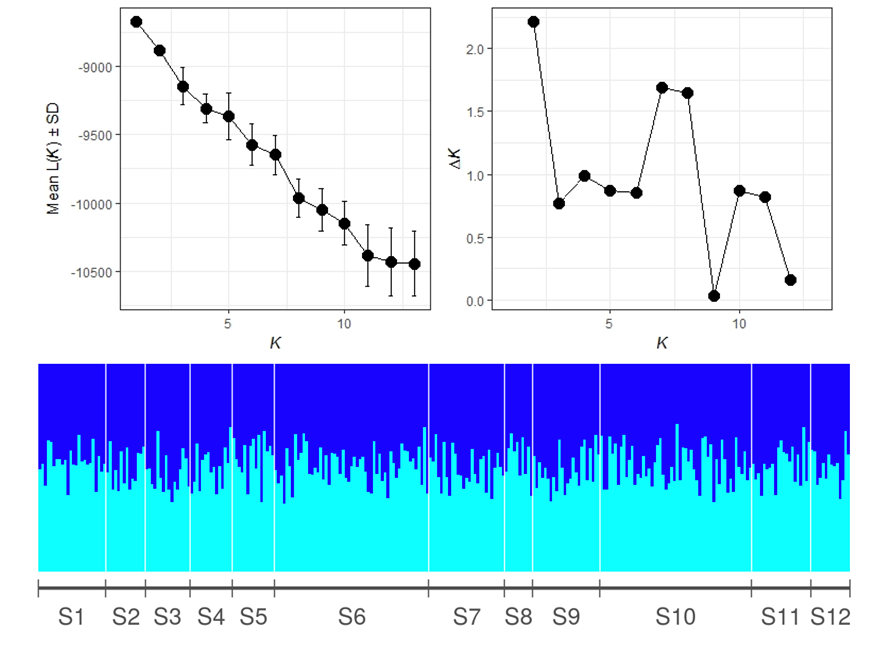

Supplement: Supplementary file 10 [file ECE3-9-3416-s010.tif]

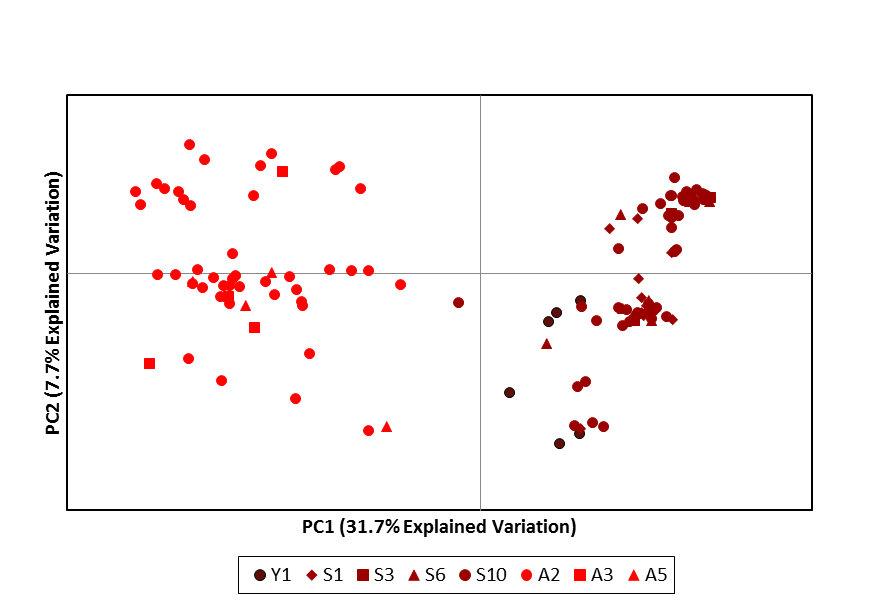

Supplement: Supplementary file 11 [file ECE3-9-3416-s011.tif]

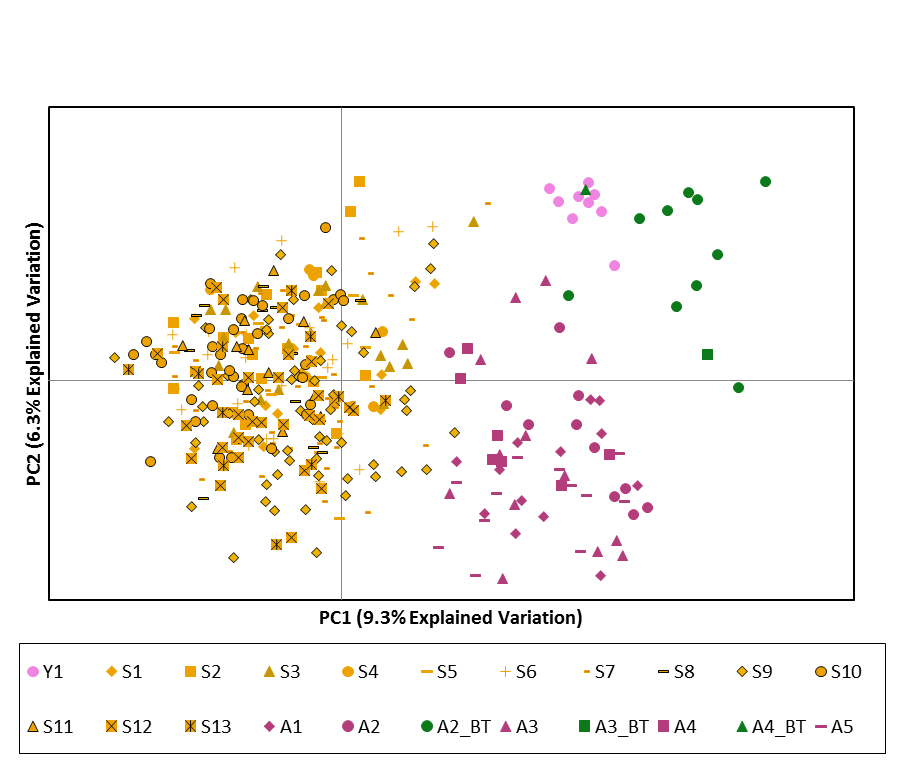

Supplement: Supplementary file 12 [file ECE3-9-3416-s012.tif]

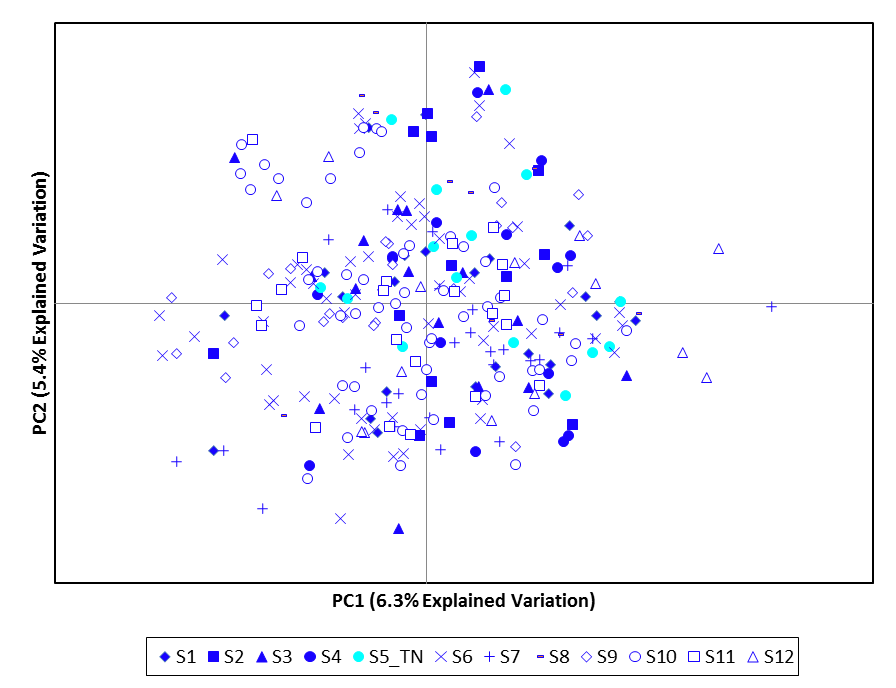

Supplement: Supplementary file 13 [file ECE3-9-3416-s013.tif]

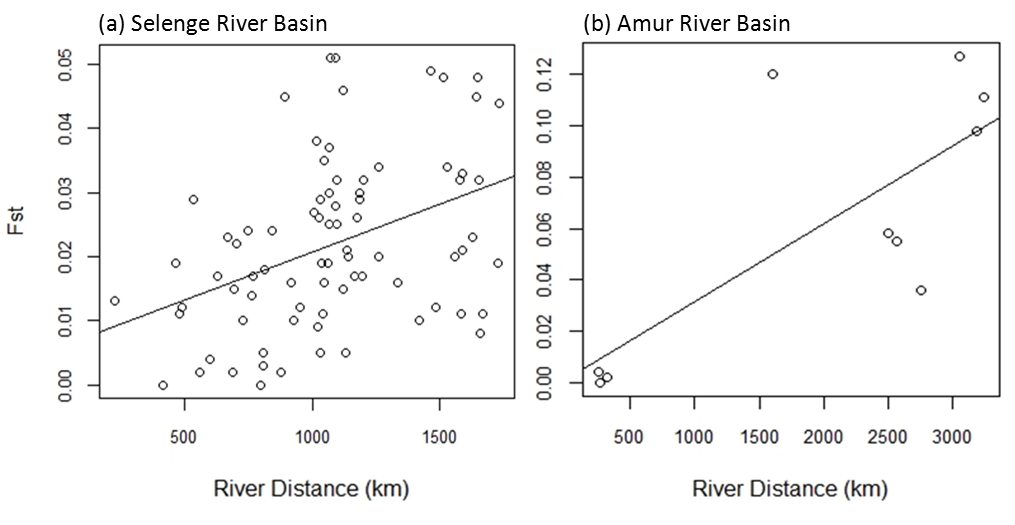

Supplement: Supplementary file 14 [file ECE3-9-3416-s014.tif]

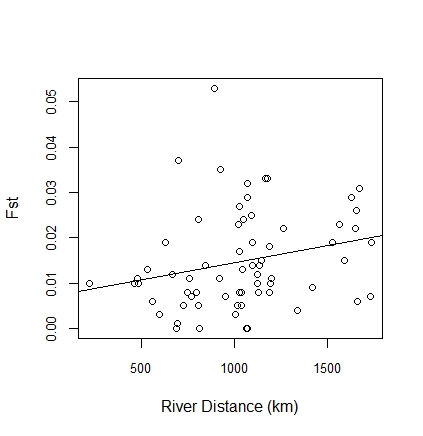

Supplement: Supplementary file 15 [file ECE3-9-3416-s015.tif]
