# Supplementary material for: Fish conservation in the land of steppe and sky: Evolutionarily significant units of threatened salmonid species in Mongolia mirror major river basins
Source: Ecol Evol. 2019 Feb 27;9(6):3416–33. doi: 10.1002/ece3.4974 (PMC6434579; doi:10.1002/ece3.4974)
Supplement: Supplementary file 16 [file ECE3-9-3416-s016.docx]

**Supplementary Material**

**mtDNA sequencing**

Sequencing was conducted in a total volume of 20 *μ*L including 5 pMol Primer LRBT-25, 10 pMol Primer LRBT-1195 (Uiblein et al. 2001), 200 *μ*M dNTP, 2 *μ*L 10x Dream Taq-buffer, 0.8 U Dream Taq-Polymerase and appr. 5 ng DNA. The PCR program involved 3 minutes at 95˚C, then 40 cycles of 95˚C for 30 seconds, 64˚C for 40 seconds, 72˚C for 1 minute and a final 10 minutes of 72˚C. PCRs products were purified by centrifuging for 3 minutes at 2800 rpm through cross linked dextran gel (Sephadex G-50 Superfine, GE Healthcare Life Sciences, Germany). PCR-products were directly cycle-sequenced using the ABI BigDye Terminater v3.1 cycle sequencing Kit using the same primers. Products were sequenced on an Applied Biosystems 3130xl Genetic Analyser (Applied Biosystems, Foster City, USA).

**Microsatellite PCR**

We used a PCR protocol with CAG/M13R-tagged forward primers and GTTT-“pigtailed” reverse primers following Schuelke (2000). Microsatellites primers were combined and amplified in either a multiplex or singleplex polymerase chain reactions (PCR) and were fluorescently labelled with FAM, NED, PET or VIC for genotyping (see Tab. S1). The PCR amplification was conducted in 5 *μ*L of 2x Qiagen Multiplex PCR kit solution, 0.6 pMol tagged (CAG/M13R) forward primer, 2.4 pMol untagged reverse primer, 2.5 pMol CAG/M13 oligo, and approximately 3 ng DNA. The PCR program conditions consisted of 15 mins at 95˚C, followed by 20 cycles of a touchdown PCR with 94˚C for 30 s, 60˚C for 30 s minus 0.5˚C per cycle, and 72˚C for 90 s; then 20 cycles of 94˚C for 30 s, 50˚C for 30 s and 72˚C for 90 s, and a final period of 10 mins at 72˚C (Faircloth et al. 2009). The PCR product was diluted 1:100 H_2_O and 2 *μ*L were added to 7 *μ*L of formamide including size standard mix (1.5 ml FA + 40 1 *μ*L LIZ 500). Samples were denatured at 95˚C for 3 minutes and placed immediately on ice. Fragment analysis was conducted using an Applied Biosystems 3130xl Genetic Analyser (Applied Biosystems, Foster City, USA) with genotyping conducted in GeneMapper 4.0 (Applied Biosystems, Foster City, USA).

**Supplementary Figure Legends**

**Figure S1a** Displays the Evanno-plots obtained from the microsatellite data analysed with STRUCTURE for all *H. taimen* sampled across eight rivers in Mongolia (Fig. 5). The largest change in Mean L(K) occurs between K=1and K=2, while the peak in ΔK is also at 2, suggesting two genetic clusters present. Each identified cluster was again run separately and both displayed K = 1 (Fig. S1 b and c).

**Figure S1b** Evanno-plots obtained from the microsatellite data analysed with STRUCTURE for all *H. taimen* sampled from the Selenge River Basin in Mongolia. Mean L(K) is highest at K=1, while the peak in ΔK is at 2, suggesting only one genetic cluster present.

**Figure S1c** Evanno-plots obtained from the microsatellite data analysed with STRUCTURE for all *H. taimen* sampled from the Amur River Basin in Mongolia. Mean L(K) is highest at K=1, while due to the low number of samples ΔK is 2, suggesting only one genetic cluster present.

**Figure S2a** STRUCTURE analysis for microsatellite data of all Brachymystax individuals collected from 19 rivers across the Yenisei (Y1), Selenge (S1 – S13) and Amur river basins (A1 – A5) in Mongolia. When all populations were included in the analysis, two genetic clusters were identified; Δ K=2. These two clusters are further analysed with the results in Fig. 6, Fig. S2 b – d.

**Figure S2 b** Following the reanalysis of the *B. lenok* “orange cluster” from the Selenge River basin (S1 – S13), L(K) is highest at K=1 and decreases with increasing K. Thus, the peak of ΔK at K=2, resulting from the Evanno et al. (2005) method is misleading. The plot of individual cluster membership for K=2 shows no biologically meaningful pattern. Overall this indicates that there was only a single cluster across the basin 🡺 K=1.

**Figure S2 c** After a separate analysis of the “green cluster”, which included *B. lenok* from the Shishged River (Y1) and Amur River basin (sites A1-A5), plus *B. tumensis* from A2, A3 and A4 populations, K=2 was identified as the most parsimonious solution, splitting blunt-snouted lenok (dark green individuals in sites A2, A3, A4) from *B. lenok*, with *B. lenok* from Y1 appearing admixed of these two groups. We consider the ΔK peak at K=5 as an artefact and biologically irrelevant. The peak is due to the low variability of L(K) at K= 5 together with the a rather large increase of L(K) from K=4 to K=5, which however is due to individual runs with particularly low L(K) at K=4. Therefore, K=2 is the most biologically meaningful solution of the genetic clustering present. This conclusion is supported by further analyses with different subsets of these two groups (see figures S3 & S4).

**Figure S2 d** The Shishged River *B. lenok* population (Y1) and *B. tumensis* individuals collected from the Onon (A2), Balj (A3) and Kherlen (A4) rivers also were clearly separated into two genetic clusters 🡺 K = 2.

**Figure S3** *B. lenok* from the Shishged River (Y1) and the Amur River basin populations (A1-A5) with all *B. tumensis* excluded, also displayed two genetic clusters; 🡺 K = 2.

**Figure S4** For *B. lenok* populations sampled from across the Amur River basin (A1 – A5) excluding all *B. tumensis* and the Shishged population, the Evanno method indicated K=2; but this is wrong as clearly, L(K) is highest at K=1 resulting in K = 1 as the most parsimonious and biologically meaningful solution 🡺 K=1.

Overall the STRUCTURE runs of *Brachymystax*, including both *B. lenok* and *B*. *tumensis* revealed four genetic clusters:

1.0. Selenge *B. lenok* (Fig. 6, S2 b)

2.1. Amur *B*. *tumensis* (Fig. 6, S2 c - d)

2.2. Shishged *B. lenok* (Fig. 6, S2 c – d, S3)

2.3 Amur *B. lenok* (Fig. 6, S2 c – d, S3, S4)

**Figure S5** STRUCTURE analysis of the microsatellite data of *Thymallus baicalensis* (including *T. nigrescens*, S5) in the Selenge River basin (S1 – S12), Mongolia, according to the Evanno et al. (2005) method only a single population was identified (K = 1). Mean L(K) is the highest solution at K=1, while ΔK is highest at K=2, however, the method is unable to detect the correct number of clusters which is K=1.

**Figure S6** Principal Component Analysis based on nuclear markers obtained from *Hucho taimen* sampled from the Yenisei River basin (Y1), the Selenge River basin (S1, S3, S6 & S10) and the Amur River basin (A2, A3 & A5) in Mongolia (n = 127).

**Figure S7** Principal Component Analysis based on nuclear markers obtained from two species from the *Brachymystax* genus sampled from the Yenisei River basin (Y1), the Selenge River basin (S1 - S13) and the Amur River basin (A1 - A5) for *B. lenok* and the Amur River basin (A2 – A4) for *B. tumensis* in Mongolia (n = 383).

**Figure S8** Principle Component Analysis based on nuclear markers obtained from *Thymallus baicalensis* sampled from across the Selenge River basin (S1 - S12) in Mongolia (n = 289). The PCA includes the putative species *T. nigrescens* from Lake Hovsgol (S5_TN).

**Figure S9** Correlation between the distance along the river and genetic distance of sampled *Brachymystax lenok* populations **(a)** Selenge River Basin (Mantel’s test statistic = 0.41, *p* = 0.004); and **(b)** Amur River Basin (excluding blunt-snouted lenok *B*. sp., Mantel’s test statistic = 0.76, *p* = 0.045).

**Figure S10** Correlation between the distance along the river and genetic distance of sampled *Thymallus baicalensis* populations (excluding *T. nigrescens* from Lake Hovsgol) in the Selenge River basin (Mantel’s test statistic = 0.24, *p* = 0.12).

**Table S1** Microsatellite primers used for *H. taimen* (HT), *B. lenok / B. tumensis* (BL / BT) and *T. baicalensis / T. nigrescens* (TB / TN) with details including repeat motif, size range of alleles in study, forward / reverse sequence, tags added to 5’ end of F primer, pigtail sequence added 5’ to R primer, florescence dye with superscript M indicating multiplex sets, Genebank accession number and source reference.

| **Locus** | **Species** | **Repeat**  **Motif** | **Range**  **of alleles** | **F / R Sequence** | **Tag*** | **Dye** | **pigtail** | **Accession No.** | **Publication** |
| --- | --- | --- | --- | --- | --- | --- | --- | --- | --- |
| BleTri2 | HT  BL/BT | (CAT)_11_ | 132-145  122-165 | CCAGGACATATTCCCTTCTAG / CCACAGCTCAGGGCAGGGAGT | CAG | VIC | GTTT | AY48448 | Froufe et al. 2004 |
| BleTri4 | HT  BL/BT | (CAT)_5_ | 106-196  94-164 | CTCCTGGAGAGGACACCACTG / CCAGCTTCCTCTGGTGGGATG | CAG | FAM | GTTT | AY48450 | Froufe et al. 2004 |
| OMM1007 | HT  BL/BT | (TCA)_15_ | 165-194  170-222 | CATCGTTTTCCTGGTTCAC / CCCTTAACTGACGCTATT | M13R | PET | GTTT | AF346669 | Jia et al. 2008 |
| OMM1011 | HT  BL/BT | (TGA)_8_ | 204-210  193-333 | CAAGGATTCGGGACAT / CACCCCTAAAGTAGAGCA | CAG | NED | GTTT | AF346672 | Jia et al. 2008 |
| OMM1105 | HT  BL/BT | (AGAC)_23_(GATA)_16_ | 109-127  116-233 | GCACACTGTCTGGGTAAGAGA / GCAGAGCCACACTAAACCA | CAG | NED | GTTT | AF352768 | Jia et al. 2008 |
| OMM1077 | HT  BL/BT | (GATA)_9_ | 292-399  291-376 | GGCTGACCAGAGAAAGACTAGTTC / TGTTACGGTGTCTGACATGC | M13R | VIC | GTTT | AF352748 | Jia et al. 2008 |
| OMM1039 | HT | (GA)_20_ | 140-179 | GGGGTAGGAGTAGACTAGACA / ATCTTTCCCTCCTTGCAC | CAG | VIC | GTTT | AF346689 | Jia et al. 2008 |
| BleTet2 | HT | (CAGA)_5_ | 139-180 | TGTCAGAGGCCTTGACTGCGT / GCTAGGCTGTTTACTCTAGGT | M13R | VIC | GTTT | AY484452 | Froufe et al. 2004 |
| BleTet5 | HT | (TGTC)_5_ | 179-182 | CTTCTTCACCCGCCTGAGTGT / TTGAATGGGCTATCTGGCTGT | CAG | FAM | GT | AY484455 | Froufe et al. 2004 |
| BleTet6 | HT | (CCTG)_7_ | 175-322 | AGACAGCATGACAGCACAACG / GGCAGACAGACAGGCAAACAG | M13R | FAM | GTTT | AY484456 | Froufe et al. 2004 |
| BleTet9 | HT | (TATC)_2_(TGTC)_12_(TATC)_3_ | 167-373 | ACTGGATAGAAAGACCTGTGG / AGATTCTTGGTAAAAGTGAAG | CAG | FAM | GTTT | AY486103 | Froufe et al. 2004 |
| BleTri3 | BL / BT | (CAT)_7_ (CATT)_7_ | 129-161 | CAGACGTGGCGCTTGTTTGGT / CTAGTCAGGAAGCAAGTGATG | M13R | FAM | GTTT | AY48449 | Froufe et al. 2004 |
| OMM1008 | BL / BT | (GAT)_11_ | 270-333 | GATCCTTTGGGAGATTAACAG / CACCACAGTTGCTACTGCC | CAG | NED | GTTT | AF346670 | Jia et al. 2008 |
| Tar100 | TB /TN | (CTTT)_23_ | 238 - 350 | TTTGGATGTGTCAGACCTG / GAGAAAGCAAGGAGAAATCAC | M13R | FAM^M2^ | GTTT | EF694937 | Diggs and Ardren 2008 |
| Tar101 | TB /TN | (CTTT)_22_ | 252-452 | CAGAGCACACCAAGCAGAG / AGGGCAAGTCATTCCAGTC | M13R | VIC^M3^ | GTTT | EF694938 | Diggs and Ardren 2008 |
| Tar103 | TB /TN | (ATCC)_7_ | 176-252 | CGGGGATCAATAAAGTATCC / CTTCACTGTCGCTGTGAGTAC | M13R | VIC^M3^ | GTTT | EF694939 | Diggs and Ardren 2008 |
| Tar108 | TB /TN | (ATAC)_27_ | 179-203 | GGGCTTTACCTGGAAACTAGC / CCATGAAATTCTTTGGAGTGG | CAG | PET | GTTT | EF694943 | Diggs and Ardren 2008 |
| Tar112 | TB /TN | (TATC)_7_ | 361-542 | CCTGGGAATCAACAAAGTATC / AGGAGGTTCAGTGAGTGTTTC | M13R | PET^M1^ | GTTT | EF694946 | Diggs and Ardren 2008 |
| Tth419a | TB /TN | (CAGA)_24_ | 107-119 | CAATTCCCTCTCAATACTTC / CACCAGCCGAGAGTC | CAG | PET^M1^ | GTTT | GU225722 | Junge et al. 2010 |
| Tth419b | TB /TN | (CAGA)_24_ | 138-162 | CAATTCCCTCTCAATACTTC / CACCAGCCGAGAGTC | CAG | PET^M1^ | GTTT | GU225722 | Junge et al. 2010 |
| Tth447 | TB /TN | (TG)_19_ | 170-206 | CTTGATTGCCATTGGATTGT / CAACATCCTTGTCGCCTCTA | M13R | FAM^M2^ | GTTT | GU225727 | Junge et al. 2010 |

* **CAG:** CAGTCGGGCGTCATCA; **M13R**: GGAAACAGCTATGACCAT

**Table S2** Pairwise estimates of *F_ST_* values for the eight populations of *Hucho taimen* sampled in the Yenisei, Selenge and the Amur River basins, Mongolia. *F_ST_* values are below the diagonal and probability (P (rand >= data) based on 999 permutations) is shown above the diagonal.

| Pop. | Y1 | S1 | S3 | S6 | S10 | A2 | A3 | A5 |
| --- | --- | --- | --- | --- | --- | --- | --- | --- |
| Y1 |  | **0.004** | **0.010** | **0.007** | **0.002** | **0.001** | **0.001** | **0.001** |
| S1 | 0.083 |  | 0.421 | 0.443 | 0.214 | **0.001** | **0.001** | **0.001** |
| S3 | 0.118 | 0.000 |  | 0.423 | 0.423 | **0.001** | **0.001** | **0.001** |
| S6 | 0.090 | 0.000 | 0.000 |  | 0.436 | **0.001** | **0.001** | **0.001** |
| S10 | 0.122 | 0.005 | 0.000 | 0.000 |  | **0.001** | **0.001** | **0.001** |
| A2 | 0.238 | 0.293 | 0.301 | 0.279 | 0.319 |  | 0.187 | **0.008** |
| A3 | 0.305 | 0.361 | 0.396 | 0.355 | 0.384 | 0.016 |  | 0.378 |
| A5 | 0.257 | 0.327 | 0.335 | 0.306 | 0.364 | 0.088 | 0.006 |  |

**Table S3** Pairwise estimates of *F*_ST_ values for the 19 populations of *Brachymystax lenok* sampled in the Yenisei, Selenge and the Amur River basins and blunt-snout lenok (*B.* sp*.*) sampled from the Amur basin only (including individuals from A2, A3 and A4). *F*_ST_ values are below the diagonal and probability (P (rand >= data) based on 999 permutations) is shown above diagonal.

|  | Y1 | S1 | S2 | S3 | S4 | S5 | S6 | S7 | S8 | S9 | S10 | S11 | S12 | S13 | A1 | A2 | A3 | A4 | A5 | *B.* sp. |
| --- | --- | --- | --- | --- | --- | --- | --- | --- | --- | --- | --- | --- | --- | --- | --- | --- | --- | --- | --- | --- |
| Y1 |  | **0.001** | **0.001** | **0.001** | **0.001** | **0.001** | **0.001** | **0.001** | **0.001** | **0.001** | **0.001** | **0.001** | **0.001** | **0.001** | **0.001** | **0.001** | **0.001** | **0.001** | **0.001** | **0.001** |
| S1 | 0.339 |  | 0.323 | 0.068 | 0.167 | 0.150 | 0.468 | **0.016** | **0.028** | **0.005** | **0.003** | 0.260 | 0.127 | **0.017** | **0.001** | **0.001** | **0.001** | **0.001** | **0.001** | **0.001** |
| S2 | 0.326 | 0.002 |  | 0.009 | 0.119 | **0.010** | 0.182 | **0.012** | **0.002** | **0.001** | **0.006** | 0.066 | **0.007** | **0.001** | **0.001** | **0.001** | **0.001** | **0.001** | **0.001** | **0.001** |
| S3 | 0.320 | 0.011 | 0.019 |  | 0.388 | 0.100 | 0.053 | **0.001** | **0.002** | **0.001** | **0.001** | 0.108 | **0.001** | **0.001** | **0.001** | **0.001** | **0.001** | **0.001** | **0.001** | **0.001** |
| S4 | 0.336 | 0.014 | 0.017 | 0.002 |  | 0.129 | 0.106 | 0.099 | **0.008** | **0.006** | **0.003** | 0.059 | **0.007** | **0.002** | **0.001** | **0.001** | **0.001** | **0.001** | **0.001** | **0.001** |
| S5 | 0.331 | 0.009 | 0.029 | 0.012 | 0.016 |  | 0.059 | **0.003** | **0.003** | **0.001** | **0.019** | 0.270 | 0.141 | **0.008** | **0.001** | **0.001** | **0.001** | **0.001** | **0.001** | **0.001** |
| S6 | 0.346 | 0.000 | 0.005 | 0.010 | 0.015 | 0.013 |  | **0.001** | **0.009** | **0.001** | **0.002** | 0.309 | **0.018** | **0.002** | **0.001** | **0.001** | **0.001** | **0.001** | **0.001** | **0.001** |
| S7 | 0.317 | 0.014 | 0.015 | 0.023 | 0.016 | 0.029 | 0.019 |  | **0.001** | **0.006** | **0.001** | **0.008** | **0.002** | **0.001** | **0.001** | **0.001** | **0.001** | **0.001** | **0.001** | **0.001** |
| S8 | 0.385 | 0.019 | 0.044 | 0.032 | 0.045 | 0.034 | 0.023 | 0.036 |  | **0.001** | **0.001** | **0.007** | **0.002** | **0.002** | **0.001** | **0.001** | **0.001** | **0.001** | **0.001** | **0.001** |
| S9 | 0.326 | 0.017 | 0.026 | 0.032 | 0.035 | 0.025 | 0.024 | 0.009 | 0.045 |  | **0.001** | **0.012** | **0.004** | **0.001** | **0.001** | **0.001** | **0.001** | **0.001** | **0.001** | **0.001** |
| S10 | 0.358 | 0.021 | 0.020 | 0.037 | 0.038 | 0.019 | 0.018 | 0.027 | 0.051 | 0.029 |  | 0.249 | **0.021** | **0.003** | **0.001** | **0.001** | **0.001** | **0.001** | **0.001** | **0.001** |
| S11 | 0.375 | 0.005 | 0.016 | 0.011 | 0.027 | 0.005 | 0.003 | 0.019 | 0.030 | 0.017 | 0.004 |  | 0.376 | 0.064 | **0.001** | **0.001** | **0.001** | **0.001** | **0.001** | **0.001** |
| S12 | 0.402 | 0.011 | 0.033 | 0.048 | 0.049 | 0.012 | 0.020 | 0.037 | 0.048 | 0.028 | 0.019 | 0.002 |  | 0.406 | **0.001** | **0.001** | **0.001** | **0.001** | **0.001** | **0.001** |
| S13 | 0.364 | 0.017 | 0.032 | 0.046 | 0.051 | 0.025 | 0.024 | 0.032 | 0.034 | 0.022 | 0.023 | 0.012 | 0.000 |  | **0.001** | **0.001** | **0.001** | **0.001** | **0.001** | **0.001** |
| A1 | 0.344 | 0.133 | 0.162 | 0.141 | 0.141 | 0.147 | 0.147 | 0.134 | 0.176 | 0.127 | 0.182 | 0.150 | 0.152 | 0.155 |  | 0.441 | 0.344 | **0.001** | **0.004** | **0.001** |
| A2 | 0.313 | 0.137 | 0.156 | 0.135 | 0.137 | 0.145 | 0.148 | 0.142 | 0.181 | 0.139 | 0.186 | 0.158 | 0.162 | 0.166 | 0.000 |  | 0.346 | **0.001** | **0.001** | **0.001** |
| A3 | 0.340 | 0.126 | 0.159 | 0.128 | 0.143 | 0.133 | 0.136 | 0.130 | 0.170 | 0.118 | 0.172 | 0.136 | 0.143 | 0.144 | 0.002 | 0.004 |  | **0.001** | **0.001** | **0.001** |
| A4 | 0.369 | 0.183 | 0.207 | 0.166 | 0.191 | 0.167 | 0.182 | 0.181 | 0.224 | 0.179 | 0.212 | 0.189 | 0.191 | 0.191 | 0.125 | 0.121 | 0.146 |  | **0.001** | **0.001** |
| A5 | 0.364 | 0.159 | 0.189 | 0.173 | 0.167 | 0.161 | 0.166 | 0.159 | 0.190 | 0.149 | 0.203 | 0.173 | 0.150 | 0.165 | 0.036 | 0.058 | 0.055 | 0.131 |  | **0.001** |
| *B.* sp. | 0.448 | 0.305 | 0.329 | 0.320 | 0.310 | 0.329 | 0.322 | 0.323 | 0.357 | 0.333 | 0.369 | 0.344 | 0.358 | 0.359 | 0.307 | 0.273 | 0.301 | 0.344 | 0.342 |  |

**Table S4** Pairwise estimates of *F*_ST_ values for the eleven populations of *Thymallus baicalensis* sampled in the Selenge River basins and *T. nigrescens* from Lake Khovsgol (S5). *F*_ST_ values are below the diagonal and probability (P (rand >= data) based on 999 permutations) is shown above diagonal.

|  | S1 | S2 | S3 | S4 | S5 | S6 | S7 | S8 | S9 | S10 | S11 | S12 |
| --- | --- | --- | --- | --- | --- | --- | --- | --- | --- | --- | --- | --- |
| S1 |  | 0.197 | **0.050** | 0.063 | **0.001** | **0.003** | 0.101 | 0.175 | **0.001** | **0.002** | 0.074 | 0.075 |
| S2 | 0.006 |  | 0.117 | 0.180 | **0.001** | **0.002** | **0.001** | 0.061 | **0.001** | **0.009** | **0.047** | 0.120 |
| S3 | 0.011 | 0.010 |  | 0.453 | **0.002** | 0.417 | **0.012** | **0.013** | 0.082 | 0.446 | 0.205 | 0.084 |
| S4 | 0.011 | 0.007 | 0.000 |  | **0.009** | 0.154 | 0.074 | **0.039** | **0.012** | 0.145 | 0.260 | 0.420 |
| S5 | 0.036 | 0.045 | 0.034 | 0.024 |  | **0.001** | **0.001** | **0.034** | **0.001** | **0.001** | **0.005** | **0.036** |
| S6 | 0.015 | 0.023 | 0.000 | 0.005 | 0.024 |  | **0.004** | **0.003** | **0.001** | 0.438 | 0.252 | **0.006** |
| S7 | 0.006 | 0.031 | 0.015 | 0.009 | 0.039 | 0.013 |  | **0.012** | **0.001** | **0.001** | **0.041** | 0.112 |
| S8 | 0.007 | 0.019 | 0.026 | 0.022 | 0.024 | 0.025 | 0.029 |  | **0.001** | **0.007** | **0.042** | **0.046** |
| S9 | 0.033 | 0.031 | 0.009 | 0.018 | 0.061 | 0.015 | 0.035 | 0.053 |  | **0.009** | **0.005** | **0.002** |
| S10 | 0.014 | 0.015 | 0.000 | 0.005 | 0.030 | 0.000 | 0.017 | 0.025 | 0.010 |  | 0.180 | **0.022** |
| S11 | 0.008 | 0.013 | 0.005 | 0.003 | 0.030 | 0.002 | 0.010 | 0.018 | 0.019 | 0.003 |  | 0.078 |
| S12 | 0.010 | 0.011 | 0.012 | 0.000 | 0.021 | 0.016 | 0.008 | 0.022 | 0.033 | 0.012 | 0.010 |  |

|  | **df** | **SS** | **MS** | **Est. Var.** | **%** | **p** |
| --- | --- | --- | --- | --- | --- | --- |
| **(a) All *H. taimen*** |  |  |  |  |  |  |
| Among Basins | 2 | 165.754 | 82.877 | 1.151 | 29% | 0.001 |
| Among Rivers within basins | 5 | 18.801 | 3.760 | 0.041 | 1% | 0.060 |
| Among Populations | 119 | 349.331 | 2.936 | 0.186 | 5% | 0.003 |
| Within Populations | 127 | 325.500 | 2.563 | 2.563 | 65% | NA |
| Total | 253 | 859.386 |  | 3.941 | 100% |  |
| **(b) Selenge *H. taimen*** |  |  |  |  |  |  |
| Among Rivers | 4 | 16.289 | 4.072 | 0.069 | 3% | 0.004 |
| Among Populations | 70 | 178.104 | 2.544 | 0.099 | 4% | 0.058 |
| Within Populations | 75 | 176.000 | 2.347 | 2.347 | 93% | NA |
| Total | 149 | 370.393 |  | 2.514 | 100% |  |
| **(c) Amur *H. taimen*** |  |  |  |  |  |  |
| Among Rivers | 2 | 11.898 | 5.949 | 0.173 | 5% | 0.008 |
| Among Populations | 49 | 171.227 | 3.494 | 0.310 | 9% | 0.002 |
| Within Populations | 52 | 149.500 | 2.875 | 2.875 | 86% | NA |
| Total | 103 | 332.625 |  | 3.358 | 100% |  |
| **(d) All *Brachymystax spp.*** |  |  |  |  |  |  |
| Among Basins | 2 | 135.780 | 67.890 | 0.492 | 15% | 0.001 |
| Among Rivers within basins | 16 | 113.269 | 7.079 | 0.100 | 3% | 0.001 |
| Among Populations | 364 | 1089.758 | 2.994 | 0.240 | 7% | 0.001 |
| Within Populations | 383 | 963.000 | 2.514 | 2.514 | 75% | NA |
| Total | 765 | 2301.808 |  | 3.346 | 100% |  |
| **(e) *B. lenok* (only)** |  |  |  |  |  |  |
| Among Basins | 2 | 124.444 | 62.222 | 0.522 | 16% | 0.001 |
| Among Rivers within basins | 16 | 96.951 | 6.059 | 0.080 | 2% | 0.001 |
| Among Populations | 352 | 1019.940 | 2.898 | 0.182 | 5% | 0.001 |
| Within Populations | 371 | 940.000 | 2.534 | 2.534 | 76% | NA |
| Total | 741 | 2181.336 |  | 3.317 | 100% |  |
| **(f) Selenge *B. lenok*** |  |  |  |  |  |  |
| Among Rivers | 13 | 114.812 | 8.832 | 0.138 | 5% | 0.001 |
| Among Populations | 301 | 860.422 | 2.859 | 0.167 | 6% | 0.001 |
| Within Populations | 315 | 795.500 | 2.525 | 2.525 | 89% | NA |
| Total | 629 | 1770.74 |  | 2.830 | 100% |  |
| **(g) Amur *B. lenok* (only)** |  |  |  |  |  |  |
| Among Rivers | 4 | 27.670 | 6.917 | 0.171 | 6% | 0.001 |
| Among Populations | 51 | 159.518 | 3.128 | 0.274 | 9% | 0.001 |
| Within Populations | 56 | 144.500 | 2.580 | 2.580 | 85% | NA |
| Total | 111 | 331.69 |  | 3.025 | 100% |  |
| **(h) All *Thymallus spp.*** |  |  |  |  |  |  |
| Among Rivers | 11 | 51.019 | 4.638 | 0.039 | 1% | 0.001 |
| Among Populations | 277 | 785.049 | 2.834 | 0.190 | 8% | 0.001 |
| Within Populations | 289 | 709.000 | 2.453 | 2.453 | 91% | NA |
| Total | 577 | 1545.067 |  | 2.682 | 100% |  |

**Table S5** Summary results for the Analyses of Molecular Variance (AMOVA) **(a)** All *H. taimen* (*F*_ST_ = 0.302), **(b)** *H. taimen* from the Selenge (*F*_ST_ = 0.027), **(c)** *H. taimen* from the Amur basin (*F*_ST_ = 0.052); **(d)** All *Brachymystax* populations including both *B. lenok* and blunt-snouted lenok (*B. sp.*; *F*_ST_ = 0.177), **(e)** *B. lenok* only (*F*_ST_ = 0.181), **(f)** *B. lenok* from the Selenge (*F*_ST_ = 0.049), and **(g)** Amur (*F*_ST_ = 0.056) basins; and **(h)** *T. baicalensis* / *T. nigrescens* (*F*_ST_ = 0.014) from the Selenge River basin.

|  |
| --- |

**Table S6** Additional genbank accessions used for inferring haplotype networks in *Hucho taimen* and *Brachymystax* spp.

| *Hucho taimen* | *Brachymystax* spp. | | | |
| --- | --- | --- | --- | --- |
| AY230447 | AY230451 | DQ017067 | EU395728 | JN680735 |
| AY230448 | AY230452 | DQ017068 | EU395729 | JN680736 |
| AY230449 | AY230453 | DQ017069 | EU395730 | JN680737 |
| AY230450 | AY230454 | DQ017070 | EU395731 | JN680738 |
| AY862343 | AY230455 | DQ017071 | EU395732 | JX227987 |
| AY862344 | AY230456 | DQ017072 | EU395733 | KC136268 |
| AY862345 | AY230457 | DQ017073 | EU395734 | KC136269 |
| AY862346 | AY230458 | DQ017074 | EU395735 | KC136270 |
| AY862347 | AY230459 | DQ017075 | EU760490 | KF647837 |
| AY862348 | AY230460 | DQ017076 | EU760491 | KF647838 |
| AY862349 | AY230461 | DQ017077 | FJ713570 | KF647839 |
| AY862350 | AY230462 | DQ017078 | FJ713571 | KF647840 |
| AY862351 | AY230463 | DQ017079 | FJ713572 | KF647841 |
| AY862352 | AY230464 | EU395717 | FJ713573 | KF647842 |
| AY862353 | AY230465 | EU395718 | FJ713574 | KF647843 |
| AY862354 | AY230466 | EU395719 | FJ713575 | KF647844 |
| AY862355 | AY230467 | EU395720 | FJ713576 | KF647845 |
| AY862356 | AY230468 | EU395721 | FJ713577 |  |
| AY862357 | AY230469 | EU395722 | FJ713578 |  |
| AY862358 | AY230470 | EU395723 | JN680730 |  |
| AY862359 | AY230471 | EU395724 | JN680731 |  |
| EU395715 | AY230472 | EU395725 | JN680732 |  |
| EU760489 | AY960113 | EU395726 | JN680733 |  |
| KF703543 | DQ017066 | EU395727 | JN680734 |  |
